# Investigation on drag reduction on rotating blade surfaces with microtextures

**DOI:** 10.3762/bjnano.15.70

**Published:** 2024-07-10

**Authors:** Qinsong Zhu, Chen Zhang, Fuhang Yu, Yan Xu

**Affiliations:** 1 College of Mechanical and Electrical Engineering, Nanjing University of Aeronautics & Astronautics, Nanjing, 210016, China

**Keywords:** blade, drag-reducing microtexture, geometrical parameters, placement position, simulations

## Abstract

To enhance the aerodynamic performance of aero engine blades, simulations and experiments regarding microtextures to reduce the flow loss on the blade surfaces were carried out. First, based on the axisymmetric characteristics of the impeller, a new simulation method was proposed to determine the aerodynamic parameters of the blade model through the comparison of flow field characteristics and simulation results. Second, the placement position and geometrical parameters (height, width, and spacing) of microtextures with lower energy loss were determined by our simulation of microtextures on the blade surface, and the drag reduction mechanism was analyzed. Triangular ribs with a height of 0.2 mm, a width of 0.3 mm, and a spacing of 0.2 mm exhibited the best drag reduction, reducing the energy loss coefficient and drag by 1.45% and 1.31% for a single blade, respectively. Finally, the blades with the optimal microtexture parameters were tested in the wind tunnel. The experimental results showed that the microtexture decreased energy loss by 3.7% for a single blade under 57° angle of attack and 136.24 m/s, which was favorable regarding the drag reduction performance of the impeller with 45 blades.

## Introduction

In order to survive, organisms in nature have undergone billions of years of evolution; their body structures have been adapted to the current environment and exhibit special functions on biological surfaces [1]. For the purpose of drag reduction, valuable inspiration can be derived from rapidly moving animals, such as the “denticles” found on the surface of shark skin, which enable high-speed swimming [2], as well as the texture of bird feathers [3]. The phenomenon of drag reduction can also be observed on the surface of plants. For example, there is a superhydrophobic structure on the surface of lotus leaves [4]. A thin gas film captured by the superhydrophobic structure creates a slip interface between gas and liquid, which effectively improves the drag reduction and antifouling performance of lotus leaves [5]. However, the structures on biological surfaces are rather complex and not directly applicable in practice. Therefore, researchers have explored the drag reduction mechanisms through replication or imitation of the microtexture found on biological surfaces; it was found that the contribution of microtextures to drag reduction primarily occurs within the boundary layer [6–7]. Lang et al. [8] constructed rectangular and sinusoidal grooves with 2 mm in width, 3 mm in depth, and 1 mm in spacing, thus mimicking the transverse grooves on the surface of dolphin skin. They observed the effect of the grooves on flow separation and boundary layer using digital particle image velocimetry. Xiao et al. [9] analyzed the drag reduction mechanism of bionic microtextures and constructed simplified V-shaped, trapezoidal, and wavy ribs by grinding. Experimental and simulation studies on aeroengine blades with such microtextures showed that the drag reduction performance of wavy ribs is better than that of the other two structures. Tian et al. [10] pointed out that, because of the complexity of microstructures on the shark skin surface, it is difficult to use a uniform method to characterize the skin surface. Triangular grooves or rectangular grooves can be used to simplify the microstructures on the shark skin surface to study the effects on hydrodynamics and aerodynamics.

**Figure 1:**
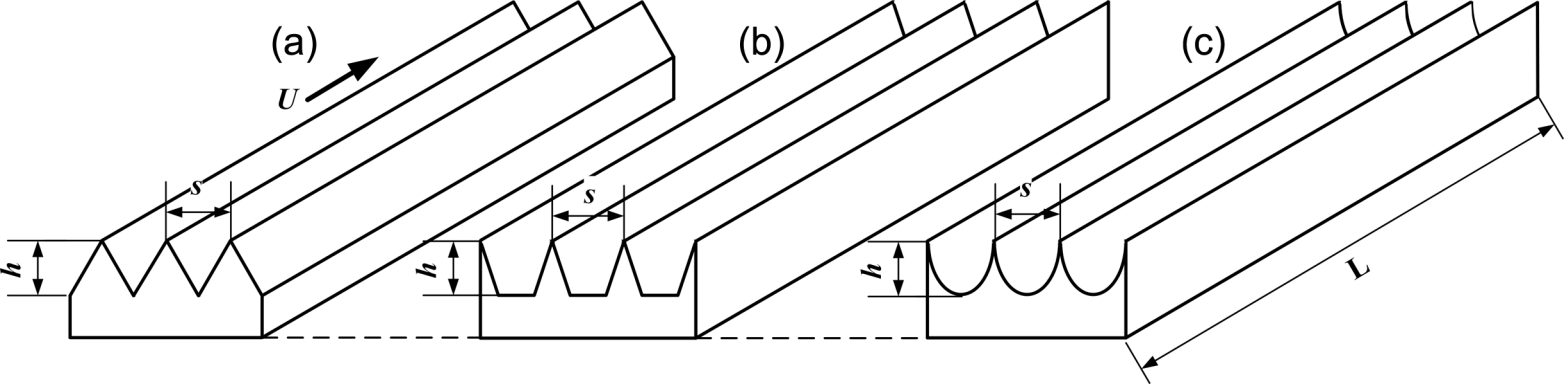
Microtextures with different sectional shapes: (a) triangles; (b) trapezoids, and (c) ellipses. The variables *s, h, L* and *U* represent the spacing, height, length, and the inflow velocity, respectively.

Within these extremely small structures, a low-speed, stable fluid flow exists, which can mitigate turbulences and enhance the stability of fluid motion within the boundary layer, resulting in a reduction of frictional drag [11]. Based on the above principles and for large-scale manufacturing, researchers imitated and simplified the microtextures of biological surfaces to form the structures with different sectional shapes, such as triangles, trapezoids, and ellipses [12–13], as shown in Figure 1.

The drag reduction effect of biomimetic microtextures can reduce friction and turbulence pulsation on blade surfaces, thus, improving the aerodynamic performance of blades [14]. The research on drag reduction of microtextures on blade surfaces can be traced back to the 1980s. In 1982, Walsh et al. [15] from NASA Langley Laboratory conducted a pioneering microtexture study on surfaces. Their experiments focused on longitudinal grooves with various shapes, revealing that symmetrical V-shaped grooves exhibited a remarkable drag reduction effect at low flow rates. The highest drag reduction rate (DRR) attained was 8%. Chamorro [16] studied fans with a grooved surface and found that, under certain operating conditions, the drag reduction effect of local coverage on the textured blade surface surpassed that of a complete covering. Additionally, they designed microgrooves of various sizes on the suction surface to achieve the optimal drag reduction effect. Zhang et al. [17] proposed a method to determine the placement position of microtextures by using finite element analysis. The suggested microtextures were arranged on blade surfaces and exhibited drag reduction compared to smooth blades. Mischo et al. [18] improved the cooling capability of turbine blades by adding grooves to the blade tips of axial turbines. Experimental and numerical simulation results showed that the addition of grooves increased turbine efficiency by 0.2% and 0.38%, respectively. In order to reduce aerodynamic losses in turbines caused by tip leakage, Parkash et al. [19] added grooves at the blade tips and verified their effectiveness through computational fluid dynamics (CFD) simulations. After the incorporation of grooves, the turbine efficiency improved by 0.1% to 0.2%.

It is evident that arranging microstructures on blade surfaces can optimize the aerodynamic performance of the blades, thereby achieving energy savings. However, the rational arrangement of microstructures on blade surfaces also requires investigation, as the shape, size, and placement position of microstructures can all affect drag reduction performance [20–21]. Wu et al. [22] investigated the effect of different sizes of triangular grooves on the drag of NACA 0012 airfoils, finding an optimal DRR of 9.65% when the microstructure dimensions were *s* = *h* = *d* = 0.1 mm. Liang et al. [23] arranged various sizes of triangular microstructures on rotating disks and found through comparative analysis that microtextures with dimensions of *s* = 0.5 mm and *h* = 0.2 mm yielded a maximum DRR of 8.46%, with the DRR varying with changes in Reynolds number. Yang and Baeder [24] designed wavy structures on the trailing edge (TE) of a wind turbine blade to reduce the recirculation flow and coherent vortex shedding; the influence of wave depth, TE thickness, and chord length on the drag force were investigated by numerical simulations. The results indicated that the maximum drag was observed at a ratio of wave depth/TE thickness = 0.25. Hossain et al. [25] constructed inward and outward dimple-like structures on the upper surface of a NACA 4415 airfoil. The results from wind tunnel experiments showed that the dimples on the airfoil surface delayed flow separation; the inward dimples increased lift by 16.43% and reduced drag by 46.66%. However, while these researches have yielded encouraging achievements in the application of microtextures on blade surfaces, achieving good drag reduction performance relies heavily on the appropriate microtextures regarding size, type, and position. The determination of such microtextures is typically impractical for the following two reasons: (1) There is a lack of analysis on the flow field over smooth blades. The phenomenon of flow separation on blades occurs because of the complex curved surface in the air flow. The placement position of microstructures needs to be determined based on the locations of flow separation on blade surfaces. (2) The analysis of drag reduction performance is mostly based on numerical simulation results, with a lack of technology research regarding the processing of microtextures and of analysis of experimental results. On the one hand, the small size of microtextures may lead to significant errors in conventional machining methods, thereby increasing the difficulty of verifying microtexture drag reduction. On the other hand, the cost associated with experiments required for microtexture testing, such as wind tunnel tests, is high.

The present study employs numerical simulations, high precision milling, and wind tunnel experiments for solving the two problems discussed above. The main contributions of this article are as follows: (1) Based on the highly symmetrical characteristics of rotating machinery such as compressors, a new numerical simulation method for blade analysis was proposed to determine the flow field on the smooth blade surface and provide references for the placement of microtextures. (2) The influence of microstructure size on drag reduction of blade surfaces was analyzed to determine the optimal parameters for microstructures. The drag reduction mechanism of the microstructures was also analyzed based on simulation results. (3) A high-precision five-axis computerized numerical control (CNC) milling machine was used to process the microtextured blades. In order to obtain high-quality surfaces on the microtextured blades, three types of end milling tools were utilized to rough- and fine-mill blade and microtextures. The microtextured blades were tested in a wind tunnel to obtain drag reduction results.

## Methods

This section introduces the modeling of microtextures on blade surfaces, as well as the equipment and processes in the actual processing of microtextures. Finally, the wind tunnel experiment platform used to measure the drag reduction of the microtextured blades is described.

### Impeller

**Figure 2:**
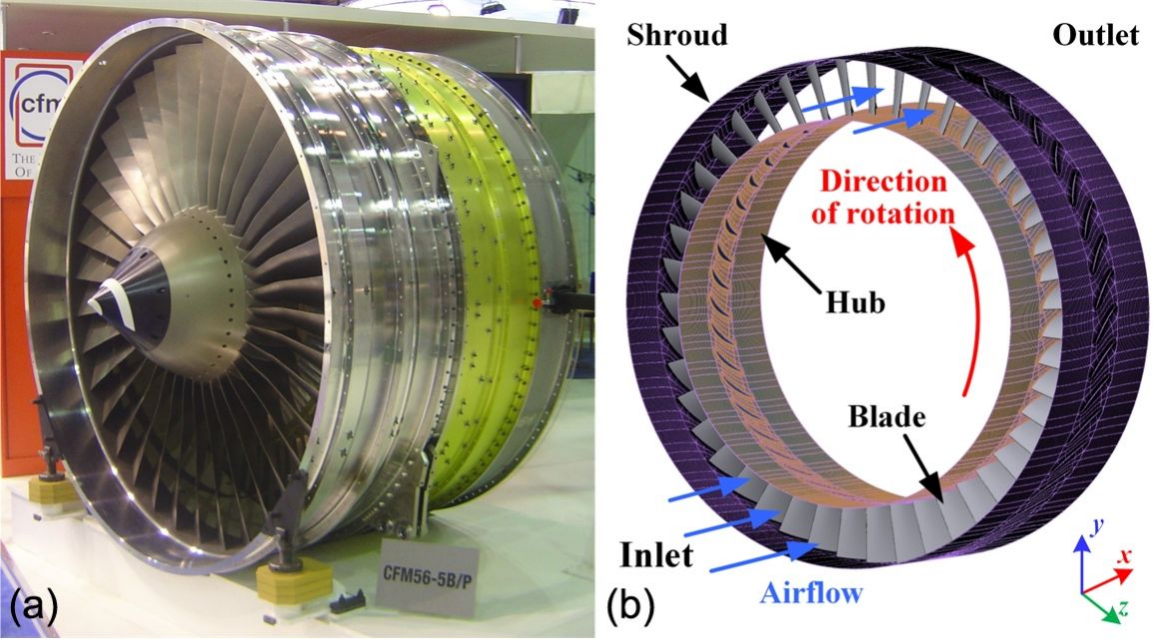
(a) Axial flow compressor [27] and (b) schematic of the impeller with 45 blades. Figure 2(a) was reproduced from [27] (Copyright © 2005 David Monniaux; “File:CFM56 dsc04641.jpg“, Wikimedia Commons, https://commons.wikimedia.org/wiki/File:CFM56_dsc04641.jpg, distributed under the terms of the Creative Commons Attribution-Share Alike 3.0 Unported License, https://creativecommons.org/licenses/by-sa/3.0/deed.en). This content is not subject to CC BY 4.0. Figure 2b is used courtesy of ANSYS, Inc.

The research object of this paper is the impeller of an axial flow compressor, which consists of a hub and blades [26]. In order to generate high pressure, axial flow compressors typically comprise multiple stages of impellers, as shown in Figure 2a. When the compressor is working, the air flow is driven by the rotation of the impeller from the inlet to the outlet, as shown in Figure 2b. The working conditions of the impeller mainly include the rotational speed and the environmental conditions (temperature and pressure) at inlet and outlet. The impeller considered in this paper has 45 blades and is designed to operate at a rotational speed of 2880 rpm. The operating environment for the impeller is at standard temperature (25 °C) and pressure (101325 Pa).

### Modeling of microtextures on blade surface

**Figure 3:**
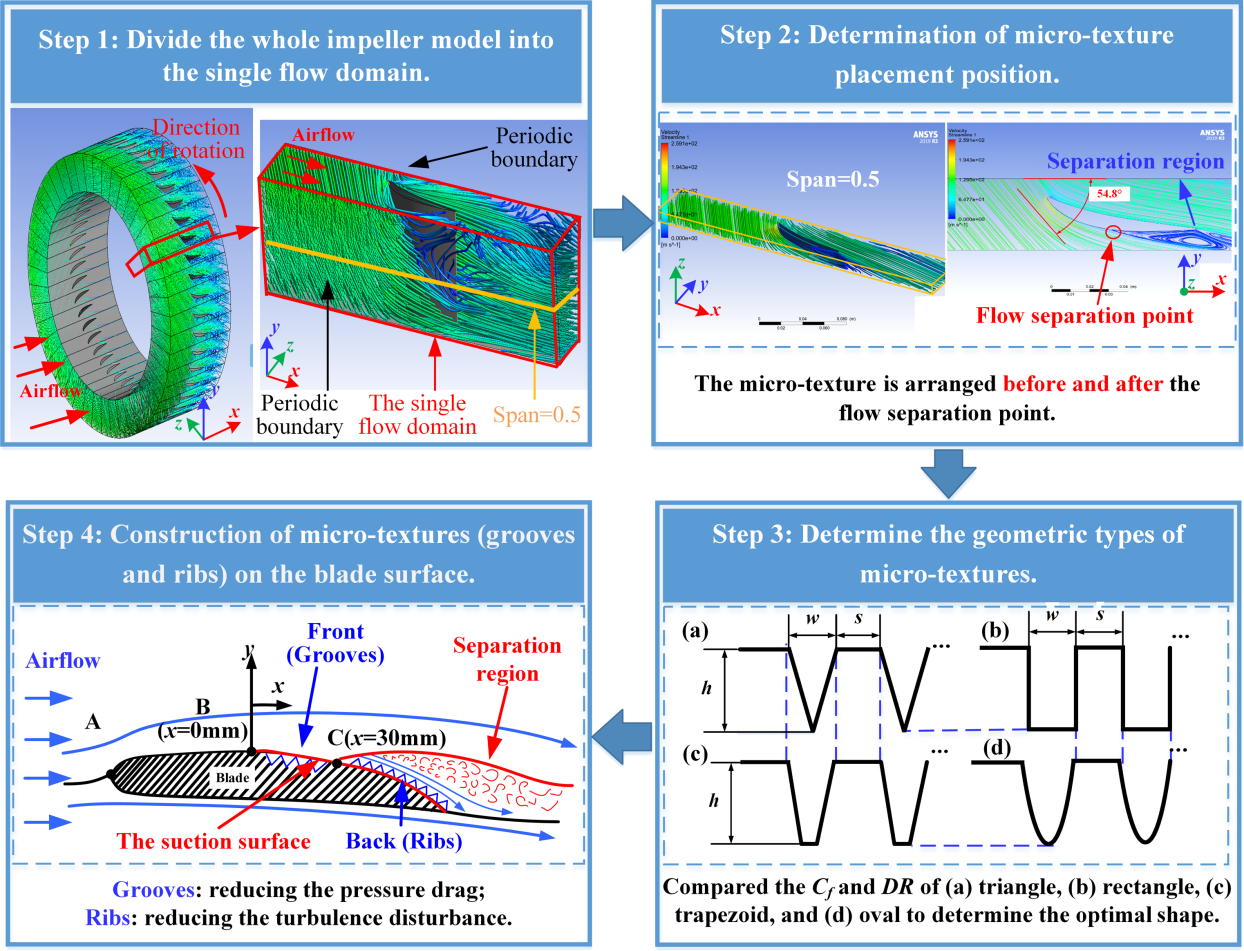
Flow chart of the simplified numerical simulation method. Images used courtesy of ANSYS, Inc.

To determine the geometry and position of the microtextures on the blade surface, a new simulation method is proposed based on the axisymmetric characteristics of rotating machinery. The complete flow domain model of the compressor was simplified into a single flow channel model, so that the flow field on the smooth blade surface could be obtained quickly and accurately. A flow diagram consisting of four steps is shown in Figure 3.

#### Step 1

The compressor model has rotational symmetry, and each blade is uniformly installed on the compressor. Therefore, the compressor model was evenly divided according to the number of blades to obtain a calculation domain model including a single blade and a single flow channel. In the simulation setup, the walls on both sides of the channels were set as periodic boundaries, which can simulate the flow domain with symmetry and make up for the calculation error caused by the simplified model. Through the above simplification, the calculation cost can be greatly reduced while ensuring calculation accuracy.

#### Step 2

The microtexture placement position is determined according to the flow field of the smooth blade. Flow separation occurs during high-speed air flow over the curved blade surface. Here, a reasonable arrangement of microtexture can effectively improve the drag reduction. Therefore, CFD was employed to simulate a single channel model of the blade and the flow separation region was obtained. In order to verify the accuracy of the simulation calculation, the theoretical calculated value of the angle of attack was compared with the simulation results.

#### Step 3

First, the drag reduction performance of four microtextures was compared by numerical simulations to determine the geometric type with the optimal drag reduction. Then, different widths (*w*), spacings (*s*), and heights (*h*) of the microtextures were compared to determine the scale range with drag reduction. In the simulation setup, the initial conditions and the flow domain are consistent with the single flow domain of the blade. The coefficient of friction and the DRR from the simulation results were compared to determine the geometric types and size ranges of the microtexture with drag reduction performance.

#### Step 4

According to the flow field on the smooth blade surface, groove and rib microtextures were arranged before and after the flow separation point on the suction surface. The difference between grooves and ribs will described in the passage referring to step 4 in the Results and Discussion section. Also, based on the microtexture types and size range determined in step 3, the drag reduction results of grooves and ribs with different size parameters were compared to determine the combination of microtexture parameters with the best drag reduction performance. This parameter combination was employed for machining microtextures on the blade surface, and the microtextured blade was placed in the wind tunnel for experiments.

### Details of step 1 and step 3

Here, the details about the simulation setup method and the determination method of microtextures in step 1 and step 3 of the proposed method are described. To enhance readability, the relevant results from steps 2, 3, and 4 will be analyzed in the Results and Discussion section.

#### Step 1

**Figure 4:**
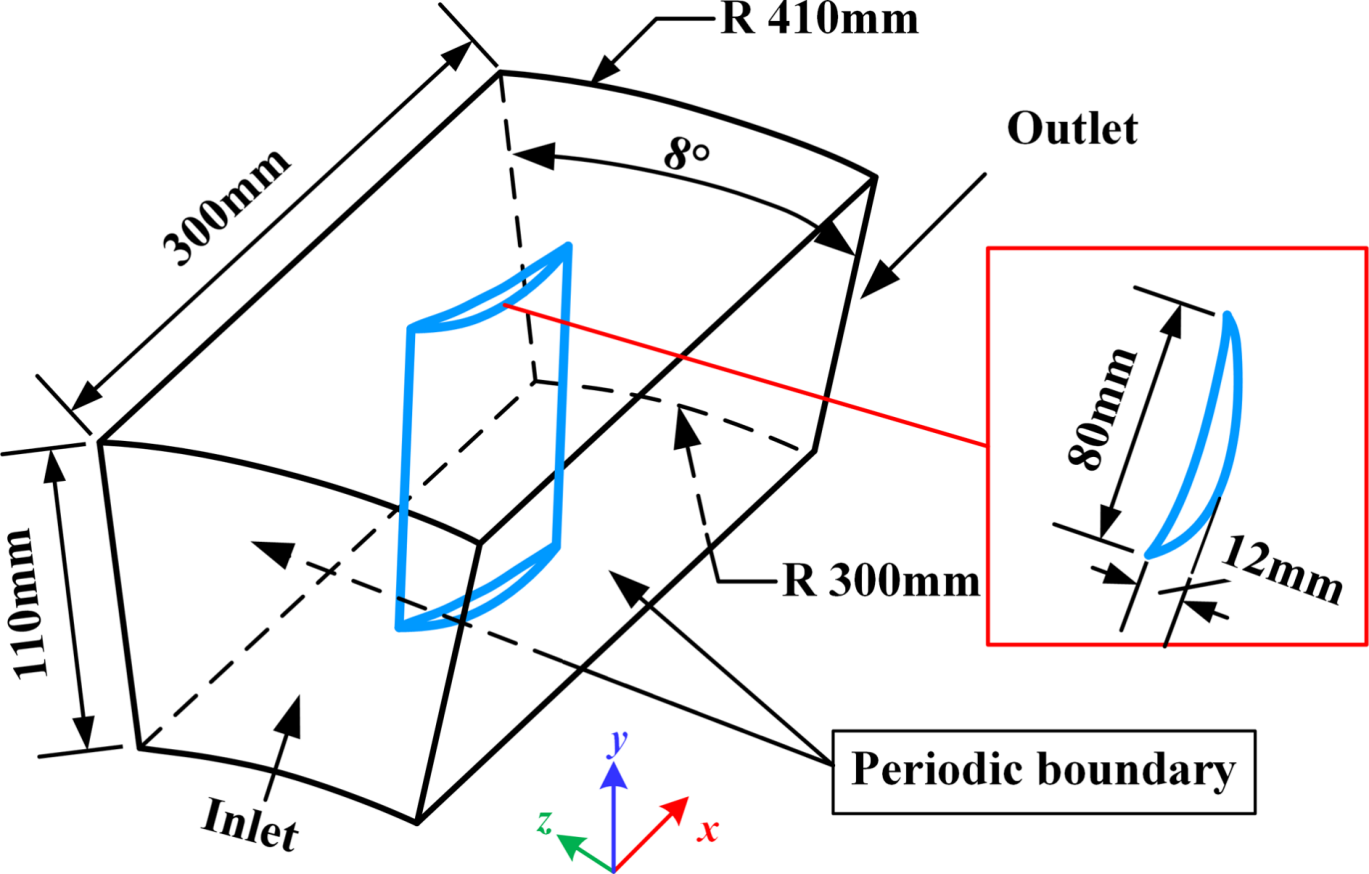
The size of flow domain and the single blade.

The whole impeller with 45 blades was divided evenly, that is, each single flow domain occupies an 8° fan-shaped flow channel, as shown in Figure 4. The length of the flow channel is 300 mm, and the bottom radius and top radii are 300 and 410 mm, respectively.

In the simulation setup, the *k*–epsilon (*k*-ε) model with enhanced wall treatment, which has better prediction results for rotation, boundary layer separation with large back pressure gradient, and backflow phenomena, was used for turbulence modeling in Ansys*®* CFX, Release 2020 R2 [28]. The boundary conditions were set according to the working conditions of the impeller, that is, the inlet of the impeller was set as the velocity inlet with a value of 75 m/s; the outlet was set as the pressure outlet with the value of 101325 Pa. Periodic boundary conditions were applied on both sides of the flow domain, and the upper and lower walls were no-slip walls, as shown in Figure 4. According to the above impeller parameters, we used the method of speed triangle [29] to calculate the theoretical value of the 0.5 blade height (span = 0.5). The relative airflow velocity was determined to be 130.67 m/s, with an angle of attack of 54.97°.

**Figure 5:**
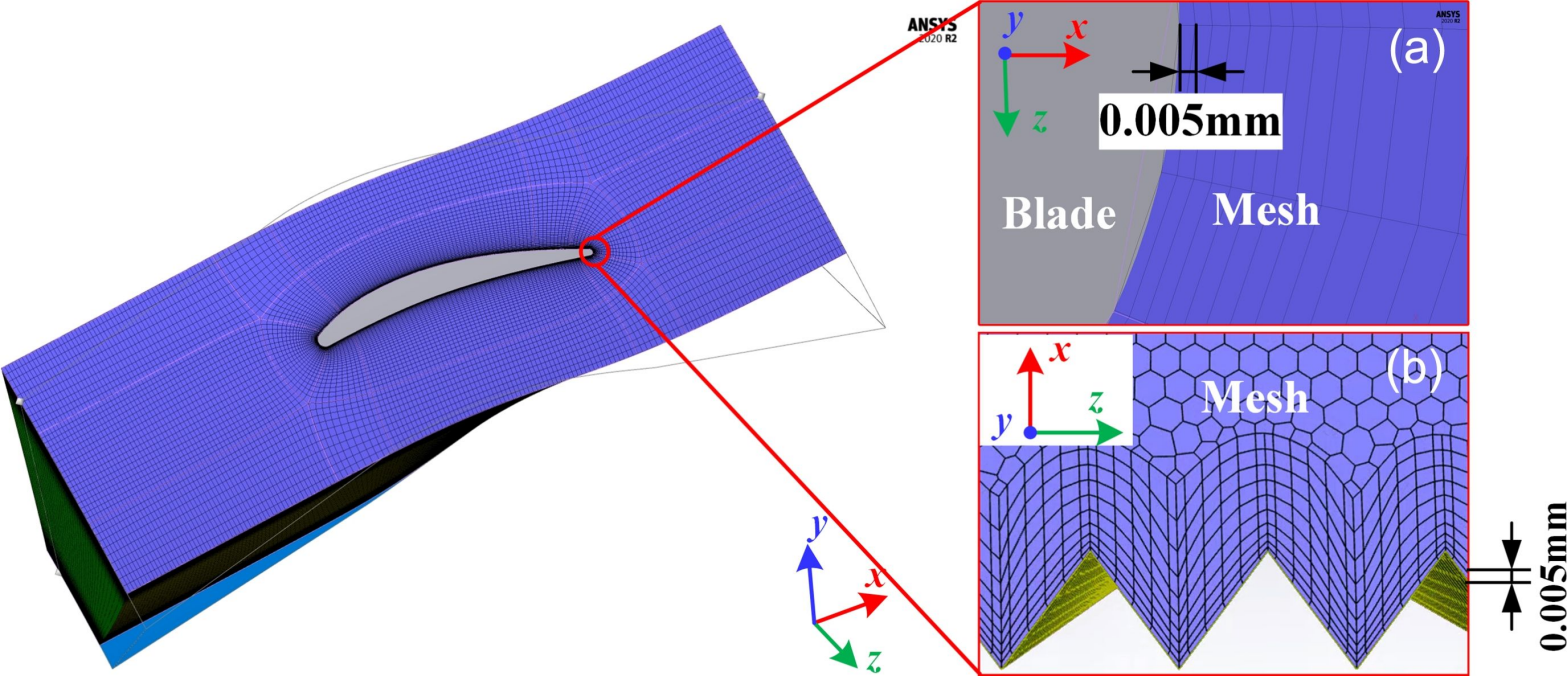
Meshed model of (a) a smooth blade surface and (b) a microtextured surface with 0.005 mm height of the first layer mesh according to *y*^+^ = 1.5. Images used courtesy of ANSYS, Inc.

The height of the first layer mesh should be calculated according to the requirement of *y*^+^ (dimensionless distance from the wall), which is determined according to each turbulence model requirements. In order to obtain good mesh quality and meet the *k-*ε model requirements used in this paper, *y*^+^ needs to be between 1 and 5 [30]. In this paper, the height of the first layer mesh is 0.005 mm, and *y*^+^ = 1.5, as shown in Figure 5.

#### Step 3

**Figure 6:**
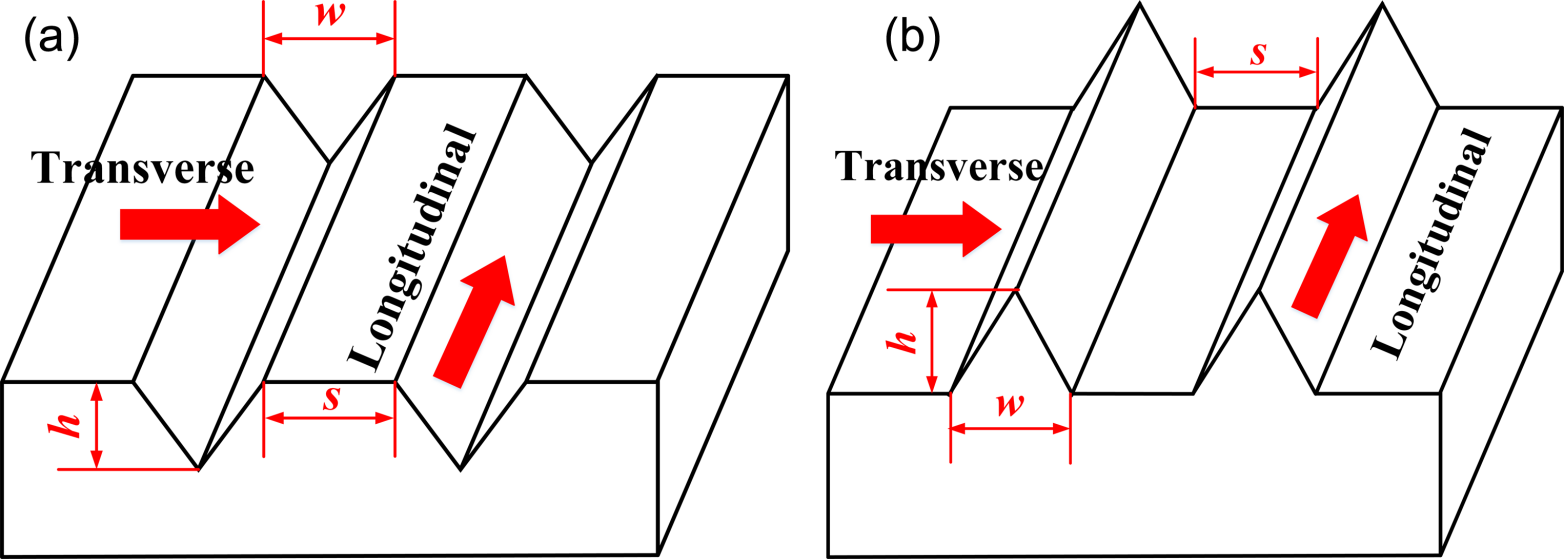
Characteristic parameters of (a) grooves and (b) ribs.

The method of dimensionless size was used to determine the size range of microtextures with drag reduction performance [31]. In this section, the microtexture sizes (i.e., *h*, *w*, and *s*) were determined according to the boundary layer theory as shown in Figure 6.

The dimensionless size calculation formula of microtextures with drag reduction performance are as follows [32]:


[1] $$s^{+}=\frac{su_{\tau }}{v},$$

[2] $$h^{+}=\frac{hu_{\tau }}{v},$$

[3] $$w^{+}=\frac{wu_{\tau }}{v},$$

[4] $$u_{\tau }={\left(\frac{\tau_{\text{w}}}{\rho }\right)}^{1/2},$$

[5] μ=ρv,

where μ is the dynamic viscosity, *v* is the kinematic viscosity, *u* is the average flow velocity, *u*_τ_ is the wall stress shear rate, τ_w_ is the wall shear stress, and ρ is the density.


[6] $$\tau_{\text{w}}=0.0225\rho u^{2}{\left(\frac{v}{u\delta }\right)}^{1/4},$$

where δ is the thickness of the boundary layer. The flow condition around the flat plate wall can be determined by the dimensionless local Reynolds number.


[7] $${\text{Re}}_{x}=\frac{\rho ux}{\mu },$$

where *x* is the distance from the inlet along the fluid flow direction. For Re*_x_* < 3 × 10^5^, the flow in the boundary layer is laminar, and the following equation yields δ_L_:


[8] $$\delta_{\text{L}}=4.96\times \left(\frac{v}{ux}\right)=4.96{\text{Re}}_{x}^{-1/5}.$$

The flow is turbulent if Re*_x_* > 3 × 10^6^, and the thickness of δ_T_ is calculated as:


[9] $$\delta_{\text{T}}=0.37\times \left(\frac{v}{ux}\right)=0.37{\text{Re}}_{x}^{-1/5}.$$

The flow in the boundary layer is transitional when Re*_x_* is between 3 × 10^5^ and 3 × 10^6^. The turbulent area is selected for the arrangement of microtextures. Therefore, Equation 9 is entered into Equation 6:


[10] $$\tau_{\text{w}}=0.029\rho u^{2}{\left({\text{Re}}_{x}\right)}^{-1/5}.$$

Entering Equation 10 into Equation 4 gives:


[11] $$u_{\tau }=0.17u{\text{Re}}_{x}^{-1/10}.$$

Entering Equation 11 into Equation 1, Equation 2, and Equation 3, respectively, yields:


[12] $$s^{+}=\frac{0.17su{\text{Re}}_{x}^{-1/10}}{v},$$

[13] $$h^{+}=\frac{0.17hu{\text{Re}}_{x}^{-1/10}}{v},$$

[14] $$w^{+}=\frac{0.17wu{\text{Re}}_{x}^{-1/10}}{v}.$$

Based on Equations 12–14, the dimensionless sizes corresponding to microtextures under different Re*_x_* can be obtained; Re*_x_* needs to be determined according to the flow velocity and the characteristic dimensions (*x*) of the calculation domain. For our blades, *u* is 75 m/s, and the maximum value of *x* is 300 mm, as shown in Figure 4. Therefore, according to Equation 13 and the range of *h*^+^ given in Table 1, the values of *h* of the microtextures with drag reduction performance were first determined. The range determination for *w* and *s* in Table 1 will be described in the Results and Discussion section.

**Table 1:** Microtexture parameters.

Parameter	Values

direction	spanwise, longitudinal
type	grooves, ribs
height (*h*)	5 < *h*^+^ < 25 [15]
width (*w*)	<3*h*
spacing (*s*)	<3*h*
position	front, back

### Experimental method

The overall process of experiments involves machining the microtextures on the blade surface and conducting experiments with the microtextured blades in a wind tunnel. The Results and Discussion section will give details about the determined microtexture types and sizes.

#### Experimental equipment

A list of equipment used in the experiment is shown in Table 2.

**Table 2:** Overview of the equipment used in the experiment.

Equipment name	Model	Purpose	Manufacturer

five-axis CNC machine tool	JDGR400-A13S	processing of blades and microtextures	Beijing Jingdiao Technology Group Co., LTD, China.
flat end mill tool	⌀8 × 37 × ⌀8 × 81 × 3F^a^	processing of blades	Shanghai Mituo CNC Equipment Co., Ltd, China.
ball end mill	⌀0.3 × 0.6 × ⌀4 × 50 × 2F^a^	roughening of microtextures	MISUMI (China) Precision Machinery Trading Co., Ltd.
ball end mill	⌀0.2 × 0.3 × ⌀4 × 50 × 2F^a^	finishing of microtextures	MISUMI (China) Precision Machinery Trading Co., Ltd.
trinocular stereo microscope	JSZ6S	observing the processed blades	Nanjing Jinsong Optical Instrument Co., Ltd, China.
three-dimensional video microscope	KH-7700	high-precision 3D imaging	QUESTAR Corporation, Japan
intermittent wind tunnel	customized equipment	aerodynamic performance testing	Nanjing Power Tiger Electromechanical Technology Co., Ltd, China.

^a^⌀(tool diameter in mm) × (cutting edge length in mm) × ⌀(shank diameter in mm) × (overall length in mm) × (number of flutes)F.

#### Microtexture processing of blade surfaces

**Figure 7:**
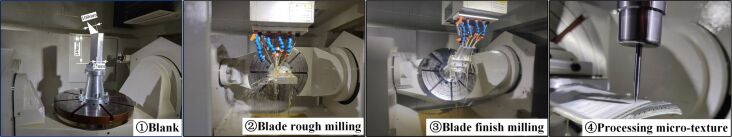
The manufacturing procedure of the microtextured blade.

A JDGR400-A13S five-axis CNC machine tool was used to process the blade and the microtextures; the processing steps are shown in Figure 7. First, the blank blade was installed in the machine tool, the size of the blank is *h* × *l* × *w* = 140 × 100 × 25 mm^3^. Second, the end milling tool was used to mill the blank roughly to improve the processing efficiency. Third, the high-quality blade models were obtained by finishing the rough blade models. Finally, the microtextures were machined on the blade surfaces; this step was also divided into roughening and finishing because the end milling tool would break if the tool with a smaller radius was used to process the microtextures directly. The tool parameters used in different processing stages are given in Table 3.

**Table 3:** Tool parameters in different machining stages.

Process stage	Tools	Tool radius (mm)	Number of flutes

roughening of blades	flat end mill	4	3
finishing of blades	flat end mill	4	3
roughening of microtextures	ball end mill	0.15	2
finishing for microtextures	ball end mill	0.1	2

#### Wind tunnel platform

**Figure 8:**
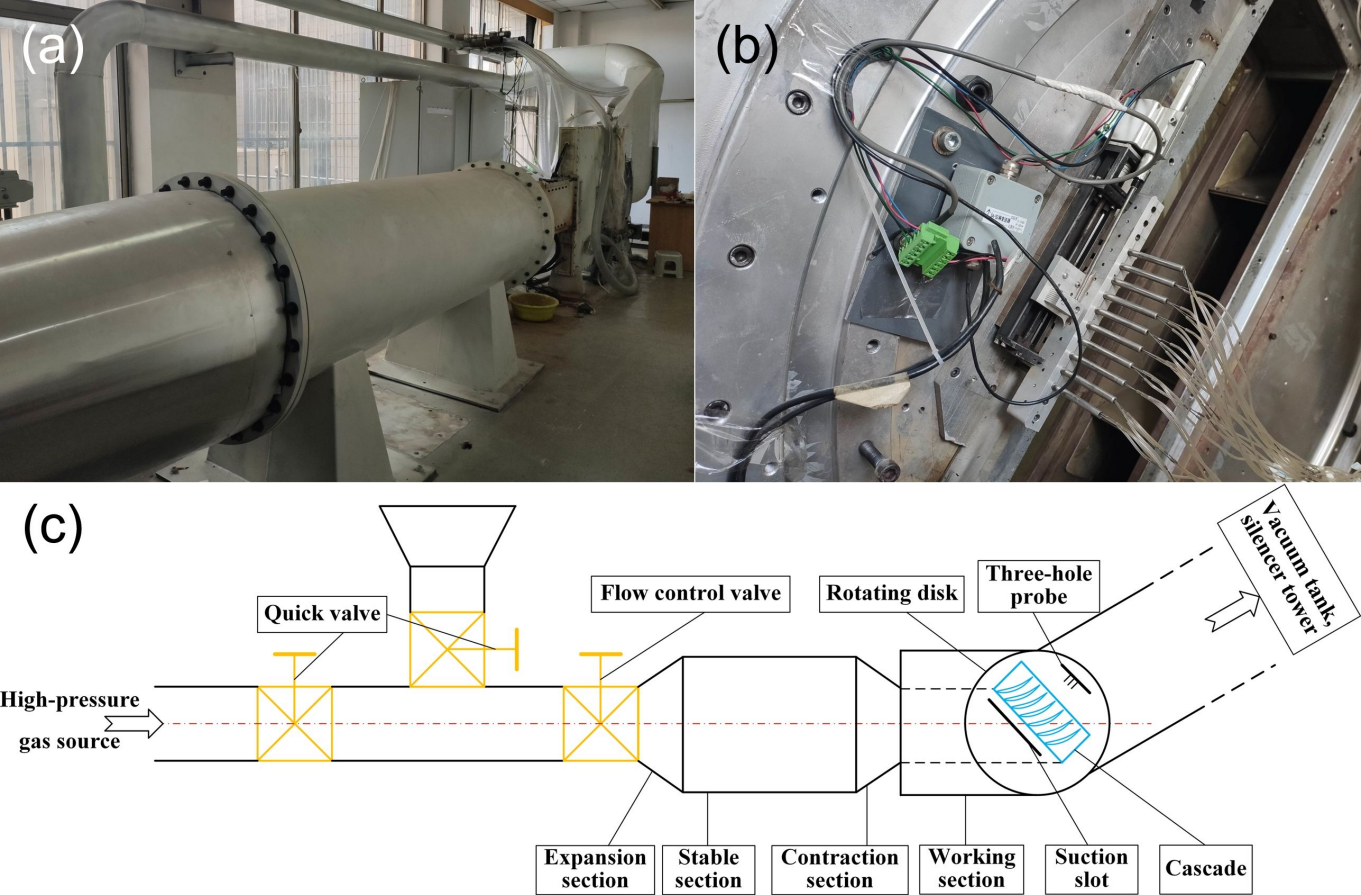
Experimental platform and schematic diagram. (a) Wind tunnel test platform. (b) Three-hole probe measuring device. (c) Schematic diagram of the wind tunnel test platform.

The experiment was conducted at the intermittent wind tunnel platform at the College of Energy and Power Engineering, Nanjing University of Aeronautics and Astronautics. Pictures and a schematic diagram of the wind tunnel test platform are shown in Figure 8. Figure 8b shows that the wake measurement device consisting of a three-hole probe, a motor, and a guide rail. Ten probes were utilized to measure the wake, while the motor facilitates control and adjustment of their position. The total pressure (*TP*), static pressure (*P*) and velocity of the air flow (*V*) in the experiment were obtained by the three-hole probe measuring device. These results can be calculated according to Equations S5–S8 in Supporting Information File 1 to obtain Mach number (Ma) and energy loss coefficient (ξ). The angle of attack can be set by controlling the motor and, thus, turning the disc (Figure 8c). In Figure 8c, the inlet of the test platform is connected with the high-pressure gas source, which is a 100 m^3^ high-pressure gas tank with a maximum of 25 atm; the air extraction source is a 200 m^3^ vacuum tank with a minimum of 0.1 atm.

#### Experimental steps

To ensure the accuracy of experiments, the velocity and angle of attack for blade heights of 0.25, 0.5, and 0.75 were selected to carry out multiple tests to verify the drag reduction effect of the microtextured blade. The specific steps of the experiment are as follows: (1) preparation of two blades in contrast, that is, one smooth blade and another blade with a microtexture on the surface; (2) test the smooth blade first; adjust the wind tunnel flow velocity and the angle of attack to 123.98 m/s and 52.8°, respectively; (3) measurement of the *TP* and *P* at the inlet and outlet, respectively, and calculation of ξ and observation of the wake loss distribution; (4) change of velocity and angle of attack to 130.67 m/s and 54.8°, respectively, and continuation according to step 3; (5) change of velocity and angle of attack to 137.54 m/s and 57.0°, respectively, and continuation according to step 3; (6) installation of the blades with microtextures and repetition of steps 3–5. The results obtained from the above steps will discussed in the “Results of the experiments” section.

## Results and Discussion

### Determined microtextures

**Figure 9:**
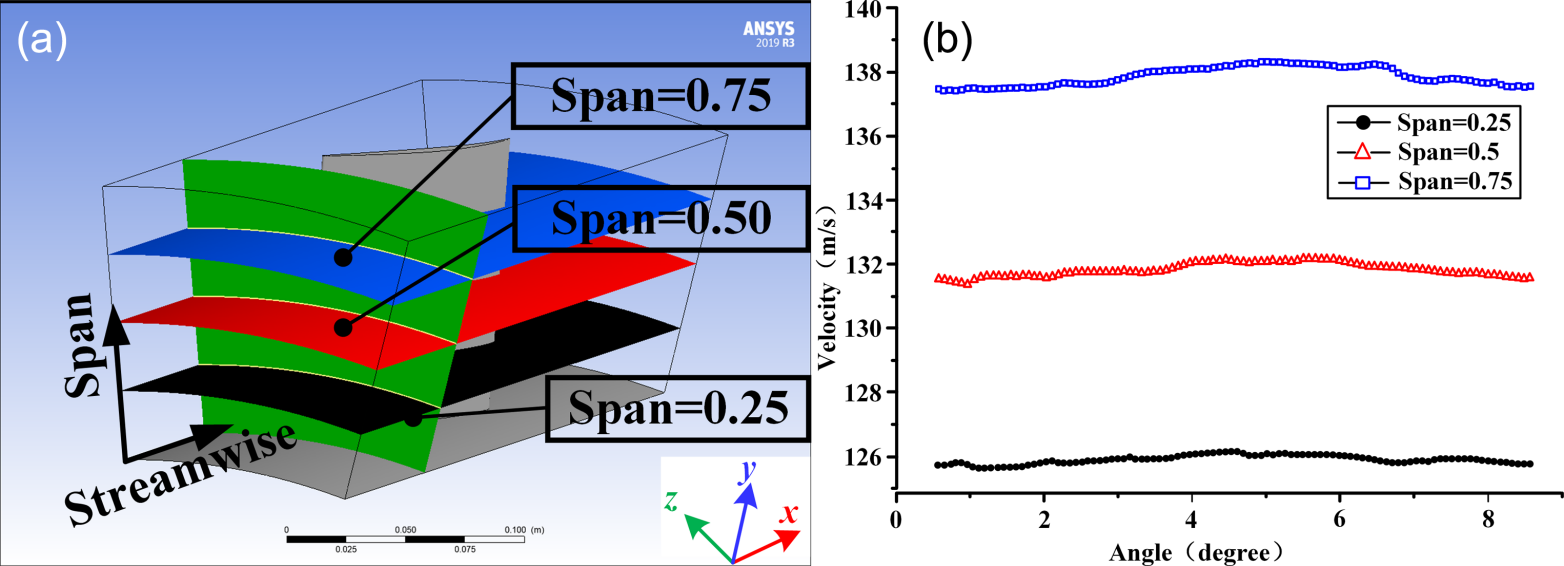
Single blade and simulation results. (a) Slices of the calculation domain. (b) Simulation results of flow velocity at span = 0.25, 0.5, and 0.75. Figure 9a used courtesy of ANSYS, Inc.

The details about the results obtained in steps 2–4 of the simulation method described in the Methods section are given here. In step 2, the simulation results of a single impeller blade were analyzed to determine the flow field characteristics around the blade, such as angle of attack, velocity, and air flow state. As shown in Figure 9a, we sliced the calculation domain to analyze the simulation results and selected the green plane at 0.25 of the total length of the flow domain in streamwise direction. In the radial direction, three curved surfaces with spans of 0.25, 0.50, and 0.75 of the blade height, progressing from the bottom to the top, were chosen for analyzing the velocity distribution at the intersection between these curved surfaces and the green plane. Figure 9b indicates an increase in the peripheral speed of the blade as the radius increases. The average velocity at span = 0.5 is 131.5 m/s, with an error of only 0.6% from the theoretical value of 130.67 m/s. This result serves as evidence supporting the reliability of the simplified simulation method; hence, the velocity in the local area simulation was set at 130.67 m/s.

**Figure 10:**
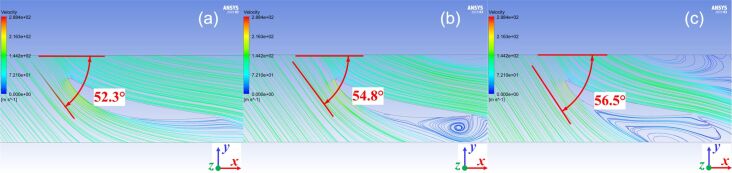
Simulation values of attack angle at (a) span = 0.25, (b) span = 0.50, and (c) span = 0.75. *x* is the axial direction, *y* is the radial direction, and *z* is the rotation direction of the blade. Images used courtesy of ANSYS, Inc.

In Figure 10, two-dimensional flow streamlines of the curved surface at span = 0.25, 0.5, and 0.75 are analyzed. The relative velocity angle of airflow and blade changes as the blades rotate. A comparison of simulation results is shown in Table 4, the error between the simulation value and the theoretical value of the angle of attack is only 0.31%. Hence, the flow field of the smooth blade surface at span = 0.5 is further analyzed.

**Table 4:** The comparison of simulated and theoretical values of the angle of attack.

Surface	Simulation values (°)	Theoretical values (°)	Error (%)

span = 0.25	52.30	52.80	0.95
span = 0.50	54.80	54.97	0.31
span = 0.75	56.50	57.00	0.88

Table 5 presents the resistance results of the smooth blade, where the total drag (*T*_d_) in the direction of airflow was divided into pressure drag (*P*_d_) and friction drag (*F*_d_). The primary impact of the microtextures is to modify the flow state of the boundary layer near the wall, reducing of *F*_d_. For our blade, the contribution of *F*_d_ is small, accounting for only 2.39% of *T*_d_. Thus, this paper primarily focuses on assessing the influence of microtextures on system energy loss.

**Table 5:** Aerodynamic parameters of the smooth blade.

Type	*P*_d_ (N)	*F*_d_ (N)	*T*_d_ (N)	*F*_d_/*T*_d_ (%)

smooth	1.3288	0.0325	1.3613	2.39

**Figure 11:**
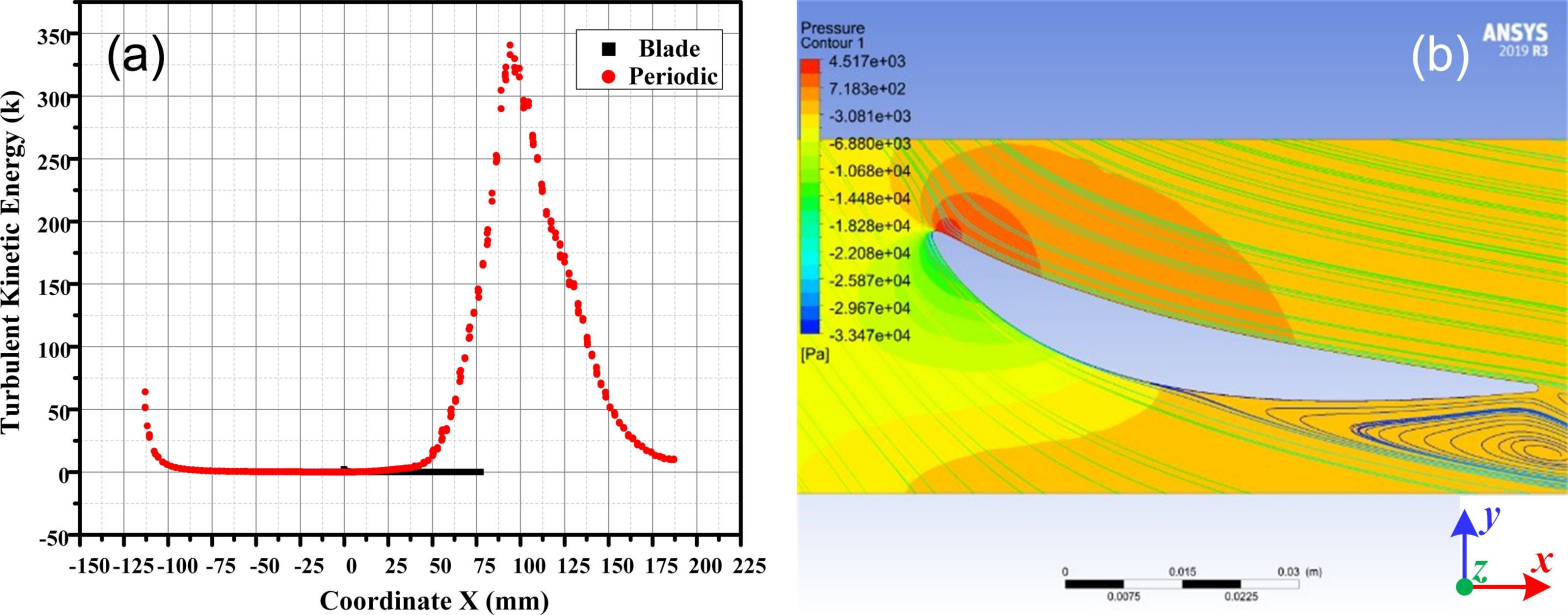
The distribution of (a) *k* and (b) the flow field on the blade surface. Figure 11b used courtesy of ANSYS, Inc.

From the leading edge to the trailing edge of the blade, the pressure surface exhibits a favorable pressure gradient, whereas the suction surface presents an adverse pressure gradient. This observation is complemented by Figure 11a, which illustrates that the turbulent kinetic energy (*k*) on the blade surface is small. The position of *X* = 0 mm in Figure 11a corresponds to the highest point of the blade surface (point B in step 4 of Figure 3). Observing the distribution of *k* on the periodic boundary shows that *k* is zero in the front region of the blade (*X* ≤ 0 mm). However, *k* begins to rise sharply from *X* = 40 mm, indicating that boundary layer separation at this position generates turbulence. The peak of *k* indicates that this position corresponds to the center of the turbulent vortex. According to Figure 11b, the turbulent vortices manifest on the adverse pressure surface within the system. Therefore, the microtextures were arranged on the adverse pressure surface.

Based on Figure 11, the air flow separation is initiated at *X* = 30 mm. As a result, the suction surface can be divided into two regions (front and back) at *X* = 30 mm, which serves as the critical point to discuss the drag reduction performance of the microtexture.

In step 3, the geometric types and size ranges of the microtextures were determined. Because of the high flow velocity on the blade surface, transverse microtextures would significantly increase the projected area in the flow direction, leading to a drastic increase in *P*_d_. Therefore, longitudinal microtextures were considered here. Because the flow projection area in longitudinal microtextures is small, *F*_d_ contributes the most to *T*_d_. *F*_d_ is related to the friction drag coefficient and the surface area, while the shape of the microtextures affects the surface area and surface flow. Therefore, microtextures of four shapes was investigated in this paper, as shown in step 3 of Figure 3.

**Figure 12:**
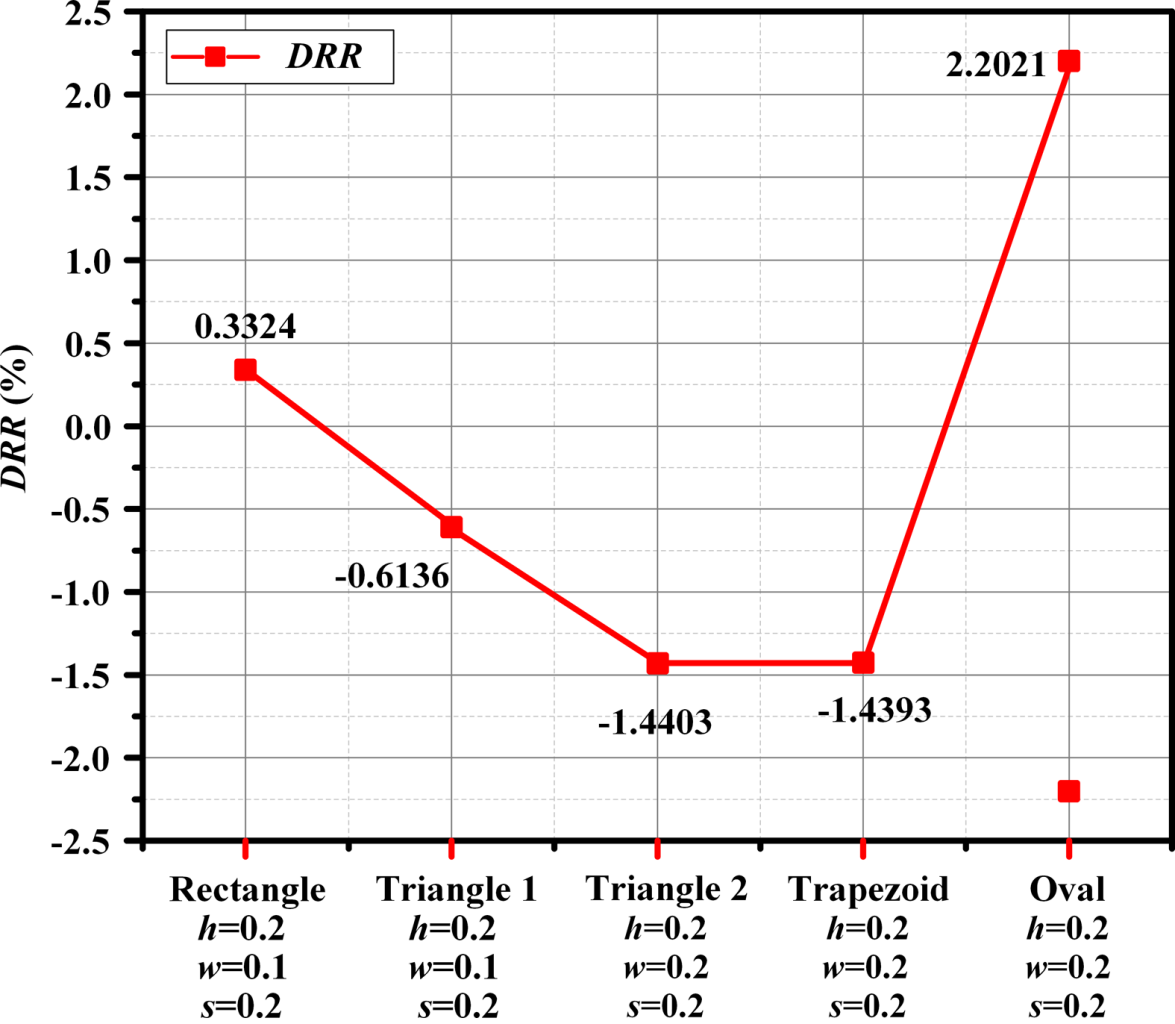
DRR results of four microtextures with different sizes. The formula for calculating DRR is shown in Equation S4 of Supporting Information File 1.

The drag reduction of the above four microtextures was simulated using the same simulation settings as in step 1. The flow velocity and the angle of attack were obtained from the results at span = 0.5, which were 130.67 m/s and 54.8°, respectively. The DRR of the four microtextures is shown in Figure 12. First, by comparing the rectangle and triangle 1 with the same values of *h*, *w*, and *s*, it is evident that the DRR of the triangle is greater than that of the rectangle. This is because the surface area of the rectangle is larger than that of triangle 1, resulting in higher frictional drag of the rectangle. Comparing triangle 2, trapezoid, and oval microtextures, it can be seen that the DRR of triangle 2 is higher than that of the other two using the same size parameters.

**Figure 13:**
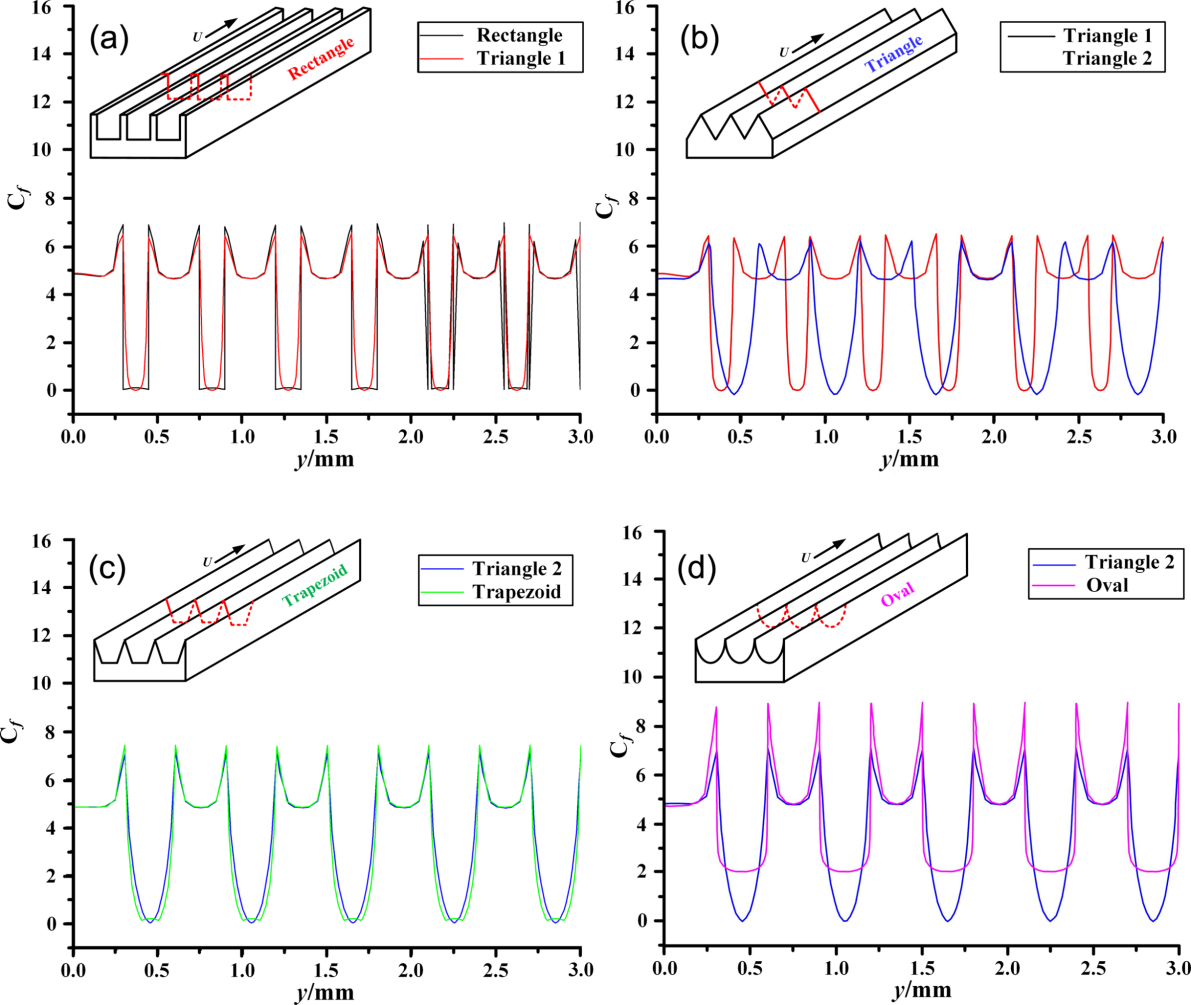
Comparison of coefficient of friction (*C*_f_) on the surface of (a) rectangle and triangle 1, (b) triangle 1 and triangle 2, (c) triangle 2 and trapezoid, and (d) triangle 2 and oval. The source of *C*_f_ is shown by the red line in the upper left corner of each panel.

Figure 13 shows the coefficient of friction (*C*_f_) of the microtexture surface. The *C*_f_ at the bottom of the microtextures is smaller because of the low speed of the fluid, which also confirms that the *C*_f_ is affected by Re. From Figure 13, it can be observed that there is a significant variation in *C*_f_ at the corners of the microtextures. Inside the grooves, *C*_f_ is smaller because the airflow velocity is lower. According to Figure 13c, there is little difference in the *C*_f_ distribution between the triangular and trapezoidal surfaces. The average values of *C*_f_ on microstructured surfaces are shown in Table 6; triangle 2 has the lowest average *C*_f_. Therefore, after comprehensive analysis of Figure 12, Figure 13, and Table 6, the triangular microstructure was chosen to be machined in the blade surface.

**Table 6:** Average values of *C*_f_.

Microtexture	Rectangle	Triangle 1	Triangle 2	Trapezoid	Oval

mean value of *C*_f_	4.323	4.2000	3.8230	3.9906	5.6677

The range of *h* has been discussed in the Methods section. In order to determine the ranges of *w* and *s*, different values of *w* and *s* of the triangular microtexture were chosen, and the DRRs were compared, as shown in Table 7 and Table 8.

**Table 7:** The influence of the *w* of the triangular microtexture on the resistance.

Group	*h* (mm)	*w* (mm)	*w*/*h*	*s* (mm)	*F*_s_ (10^−5^ N)^a^	*F*_m_ (10^−5^ N)^b^	DRR (%)

W1	0.2	0.1	0.5	0.3	5.957	5.930	−0.4459
W2	0.2	0.15	0.75	0.3	6.689	6.633	−0.8353
W3	0.2	0.2	1.0	0.3	7.415	7.317	−1.3114
W4	0.2	0.3	1.5	0.3	8.853	8.673	−2.0360
W5	0.2	0.4	2.0	0.3	10.300	10.181	−1.1593
W6	0.2	0.6	3.0	0.3	13.090	13.192	0.0147

^a^*F*_s_ is the total resistance of the smooth wall from the simulation results; ^b^*F*_m_ is the total resistance of the microtextured wall from the simulation results.

**Table 8:** The influence of the *s* of the triangular microtexture on the resistance.

Group	*h* (mm)	*w* (mm)	*s* (mm)	*s*/*h*	*F*_S_ (10^−5^ N)^a^	*F*_m_ (10^−5^ N)^b^	DRR (%)

S1	0.2	0.3	0	0	4.361	4.121	−5.5138
S2	0.2	0.3	0.10	0.50	5.866	5.683	−3.1191
S3	0.2	0.3	0.15	0.75	6.613	6.427	−2.8093
S4	0.2	0.3	0.20	1.00	7.359	7.172	−2.5400
S5	0.2	0.3	0.30	1.50	8.853	8.673	−2.0360
S6	0.2	0.3	0.40	2.00	10.346	10.167	−1.7255
S7	0.2	0.3	0.60	3.00	13.333	13.155	−1.3335

^a^*F*_s_ is the total resistance of the smooth wall from the simulation results; ^b^*F*_m_ is the total resistance of the microtextured wall from the simulation results.

Table 7 shows the change of DRR for different *w* when *h* and *s* of the triangular microtexture are fixed values. It can be clearly seen that the microtexture increases the resistance (DRR < 0) when *w*/*h* = 3, and the microtexture has the best drag reduction performance when *h* = 0.2 mm, *w* = 0.3 mm, and *s* = 0.3 mm. Therefore, one of the requirements for the triangular microtexture with drag reduction is to meet the condition of *w*/*h* < 3. Table 8 shows the influence of different *s* on the DRR. The maximum value of DRR is −5.5138% when *s* = 0 of the triangular microtexture, and the DRR gradually decreases with the increase of *s*. The triangular microtexture exhibits drag reduction under the condition of *s*/*h* < 3. In summary, the microtexture under the flow conditions described in this paper exhibits drag reduction only when *w*/*h* < 3 and *s*/*h* < 3.

In step 4, construction and comparison of different microtextures were carried out. To explore the effect of the microtextures on flow field and resistance, the grooves and ribs were arranged at the front and back sections of the suction surface, respectively, as shown in step 4 of Figure 3. According to the flow field information from the smooth blade, the velocity in the front section of the suction surface is faster. The placement of ribs here increases the projection area, resulting in the increase of *P*_d_, which will lead to advanced transition and separation of the flow; hence, the groove structure needs to be arranged in the front section. In contrast, the back section of the suction surface already exhibits separated boundary layers and turbulent vortices, and the ribs closer to the vortex have a more significant impact on the flow of the vortex. The ribs were arranged in the back section of the blade suction surface to optimize the lifting effect on the vortex. The results of drag reduction performance of microtextured surfaces are shown in Table 9.

**Table 9:** Simulation results of different types of microtexture in different regions on the blade suction surface.

Group	Region	Type	*h* (mm)	*w* (mm)	*s* (mm)	DRR (%)	η_ξ_ (%)^a^

case 1	front	grooves	0.1	0.1	0.1	0.23	1.70
case 2	front	grooves	0.2	0.2	0.2	6.52	26.88
case 3	front	grooves	0.3	0.3	0.3	18.36	42.13
case 4	back	ribs	0.1	0.1	0.1	−1.31	−1.37
case 5	back	ribs	0.2	0.2	0.2	−1.17	−1.43
case 6	back	ribs	0.3	0.3	0.3	−1.16	−1.09

^a^The change rate of energy loss coefficient from Equation S5 in Supporting Information File 1.

Table 9 shows that adding microtexture changes the force on the blade. Compared with the back section of the suction surface, the drag increase and loss coefficient changes caused by the microtextures in the front section are more pronounced. Case 1 to case 3 indicate an increase in system energy loss without drag reduction effect. Moreover, a linear relationship exists between the rate of drag change and the height of the microtexture as the height directly influences the projected area and surface area. Ribs located in the back section of the blade exhibit a drag reduction effect, which is independent of the rib height.

**Figure 14:**
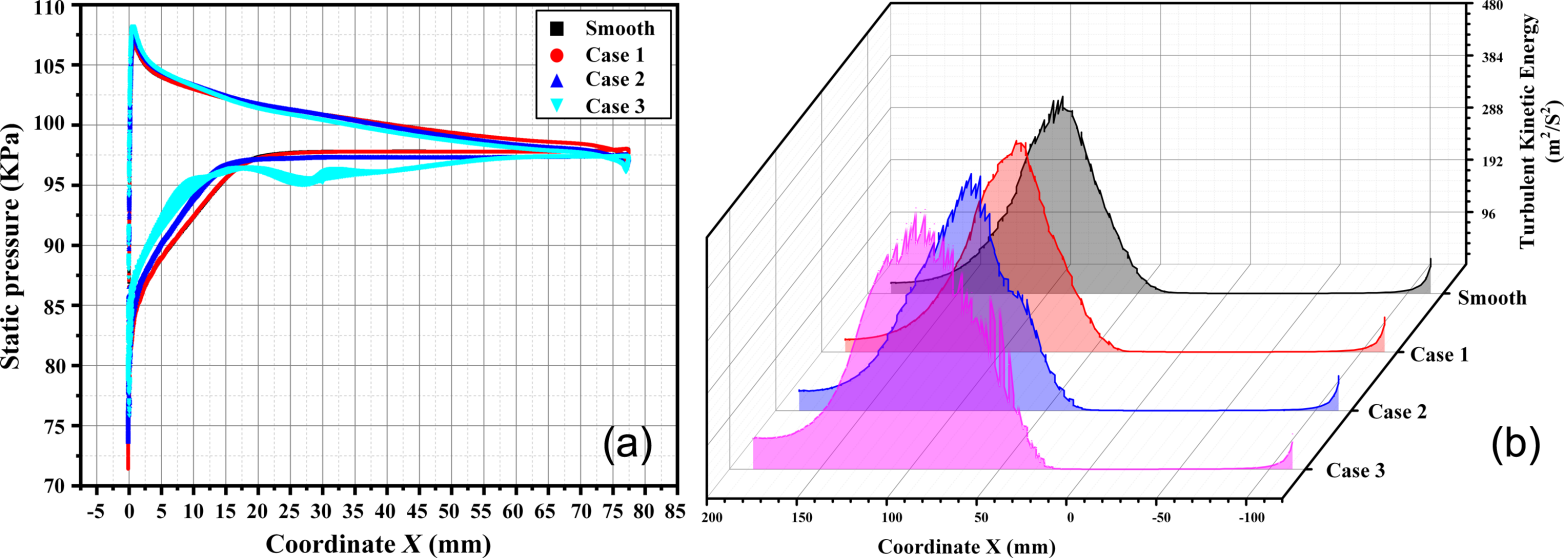
(a) Static pressure and (b) turbulent kinetic energy distribution of blade surfaces with different groove parameters.

The surface pressure distribution at the front end of the textured blade is shown in Figure 14a. It is evident that grooves substantially influence the pressure distribution, with more significant impact observed as the height of the microtexture increases. Conversely, the pressure distribution trend of the groove surface with a height of 0.1 mm resembles that of a smooth surface. Therefore, the groove has little effect on drag and ξ.

According to Figure 14b, the grooves arranged in the front section of the blade suction surface lead to an increase in turbulent kinetic energy. This results in an earlier increase in turbulence intensity, indicating the premature separation of the boundary layer and an associated increase in energy loss. From both the perspective of resistance changes and energy loss, grooves do not effectively contribute to drag reduction performance.

In Table 9, the microtextures were arranged at the back of the blade suction surface, which explicitly affects the DRR and ξ. In order to explore the drag reduction characteristics of ribs on the back, the rib parameters were further analyzed. The specific simulation results are shown in Table 10.

**Table 10:** Simulation results of ribs with different parameters arranged at the back section of the blade surface.

Group	Region	Type	*h* (mm)	*w* (mm)	*s* (mm)	DRR (%)	η_ξ_ (%)^a^

case 4	back	ribs	0.1	0.1	0.1	−1.31	−1.37
case 5	back	ribs	0.2	0.2	0.2	−1.17	−1.43
case 6	back	ribs	0.3	0.3	0.3	−1.16	−1.09
case 7	back	ribs	0.2	0.1	0.2	−1.30	−1.13
case 8	back	ribs	0.2	0.3	0.2	−1.31	−1.45
case 9	back	ribs	0.2	0.3	0	−1.19	−1.23
case 10	back	ribs	0.2	0.3	0.1	−1.15	−1.09

^a^The change rate of energy loss coefficient from Equation S5 in Supporting Information File 1.

**Figure 15:**
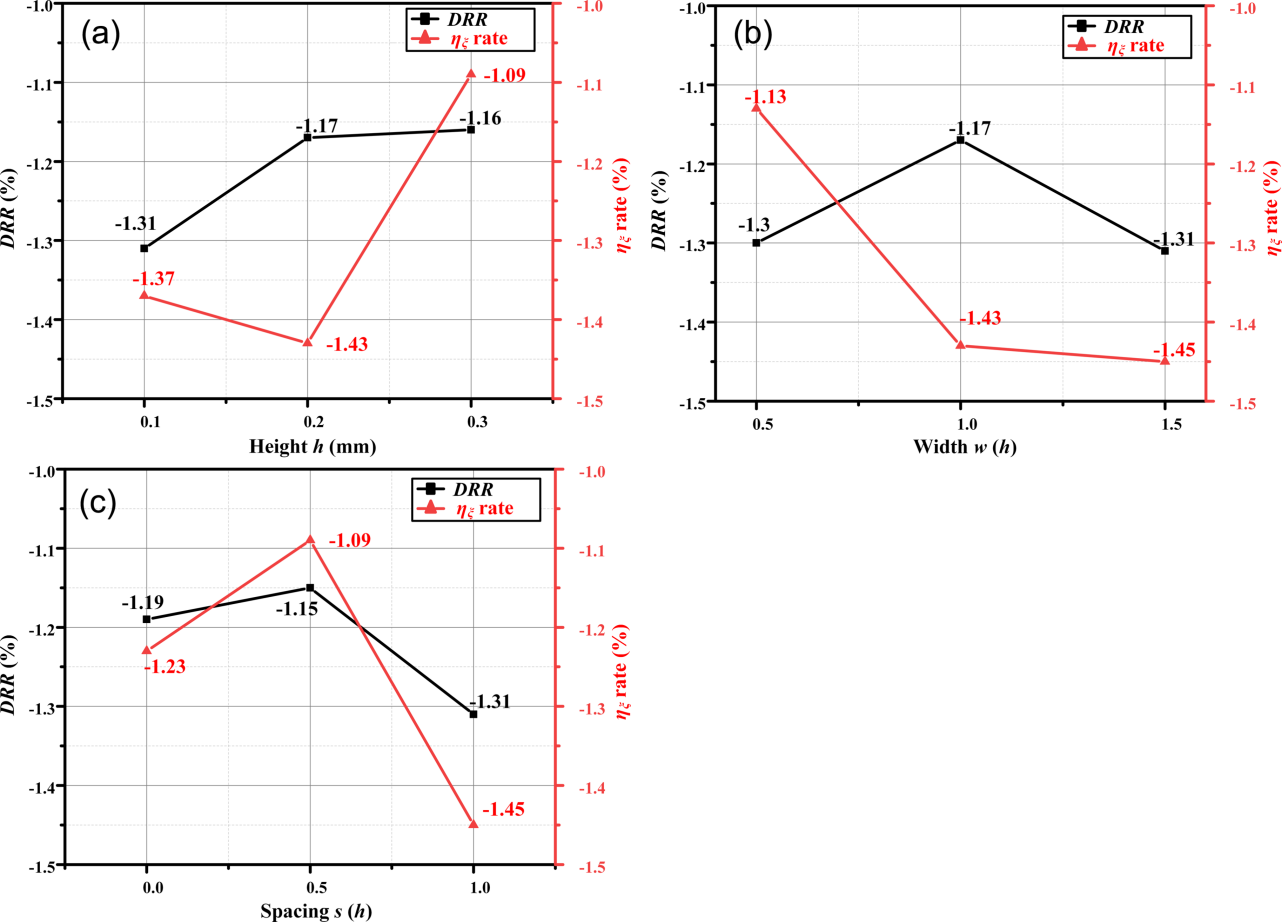
Influence of (a) height, (b) width, and (c) spacing of microtextures on drag reduction performance.

As shown in Figure 15a, the resistance increases with the height of the ribs. However, this relationship is not linear and is influenced by the coupling effect of the microtexture on *P*_d_ and *F*_d_. The highest DRR of −1.31% is obtained when *h* = 0.1 mm, while the lowest energy loss coefficient is observed when *h* = 0.2 mm. As shown in Figure 15b, the DRR is the highest when *w* = 1.5*h*, basically the same as for *w* = 0.5*h*, and the energy loss coefficient is the highest when *w* = 1.5*h*. We therefore select cases 8–10 (*w* = 1.5*h*) to research the spacing parameter further. According to Figure 15c, the spacing exhibits the same effect on the DRR and energy loss coefficient. Moreover, the drag reduction effect is optimal when *s* = *h*.

The DRR alone cannot fully represent the overall energy consumption for the entire impeller system. Thus, we comprehensively consider the two simulation results to guide the microtexture design. The selection criterion is based on achieving the smallest energy loss coefficient and the highest DRR. Following this standard, ribs with *h* = 0.2 mm, *w* = 0.3 mm, and *s* = 0.2 mm (case 8) exhibit the best performance. The maximum DRR and η_ξ_ = are −1.31% and −1.45%, respectively, in case 8, and the drag reduction effect is significant for the whole impeller system with 45 blades.

### Drag reduction mechanism analysis

**Figure 16:**
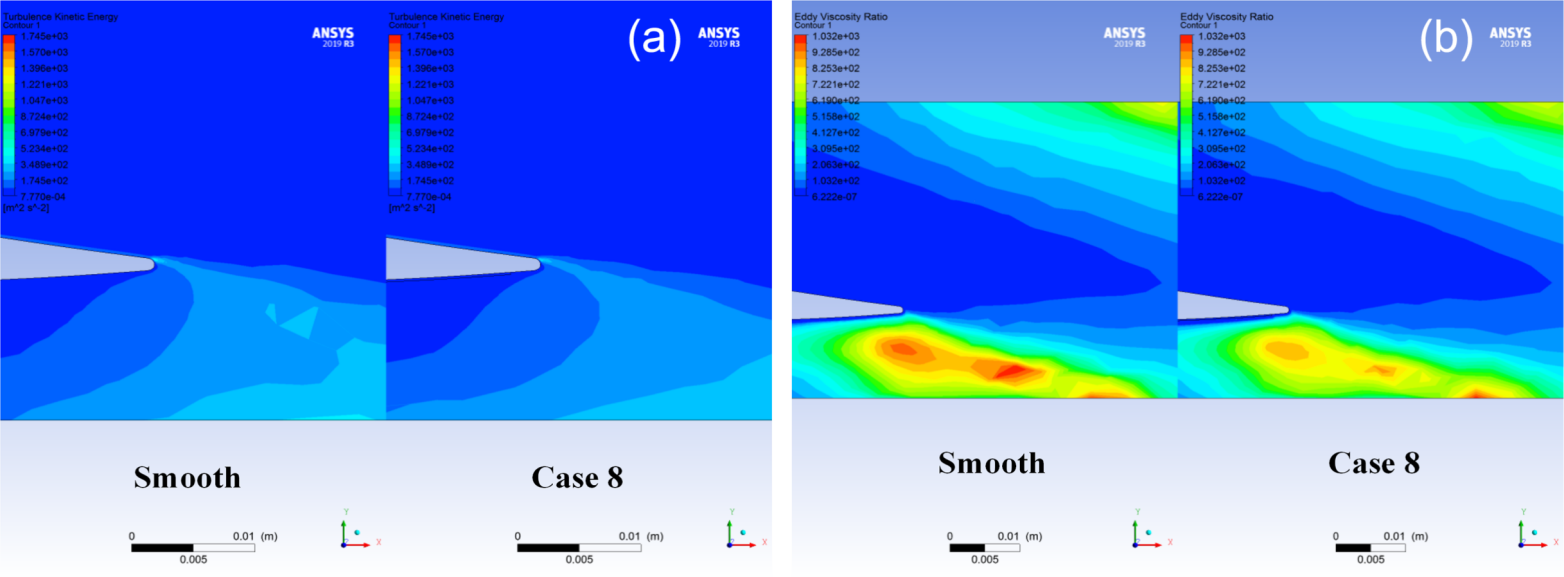
The effect of microtexture on (a) turbulent kinetic energy and (b) eddy viscosity ratio around blades. Images used courtesy of ANSYS, Inc.

The effective method to reduce drag in the flow field is to delay boundary layer separation and inhibit turbulence generation [33]. Because turbulence generation leads to energy dissipation, increasing the energy loss. Therefore, the drag reduction of the microtextured blade surface was analyzed by considering turbulent kinetic energy, eddy viscosity ratio, and flow field. Figure 16a compares smooth blades and textured blades (case 8) regarding the turbulence in the surrounding flow field. The presence of the microtexture on the blade surface results in a decrease in turbulent kinetic energy at the back end of the blade, thereby reducing energy losses. Figure 16b compares the eddy viscosity ratio, representing the stress generated by turbulent motion. The microtexture significantly reduces the stress generated by turbulent motion. As a result, the energy loss in the entire flow channel system is substantially reduced.

**Figure 17:**
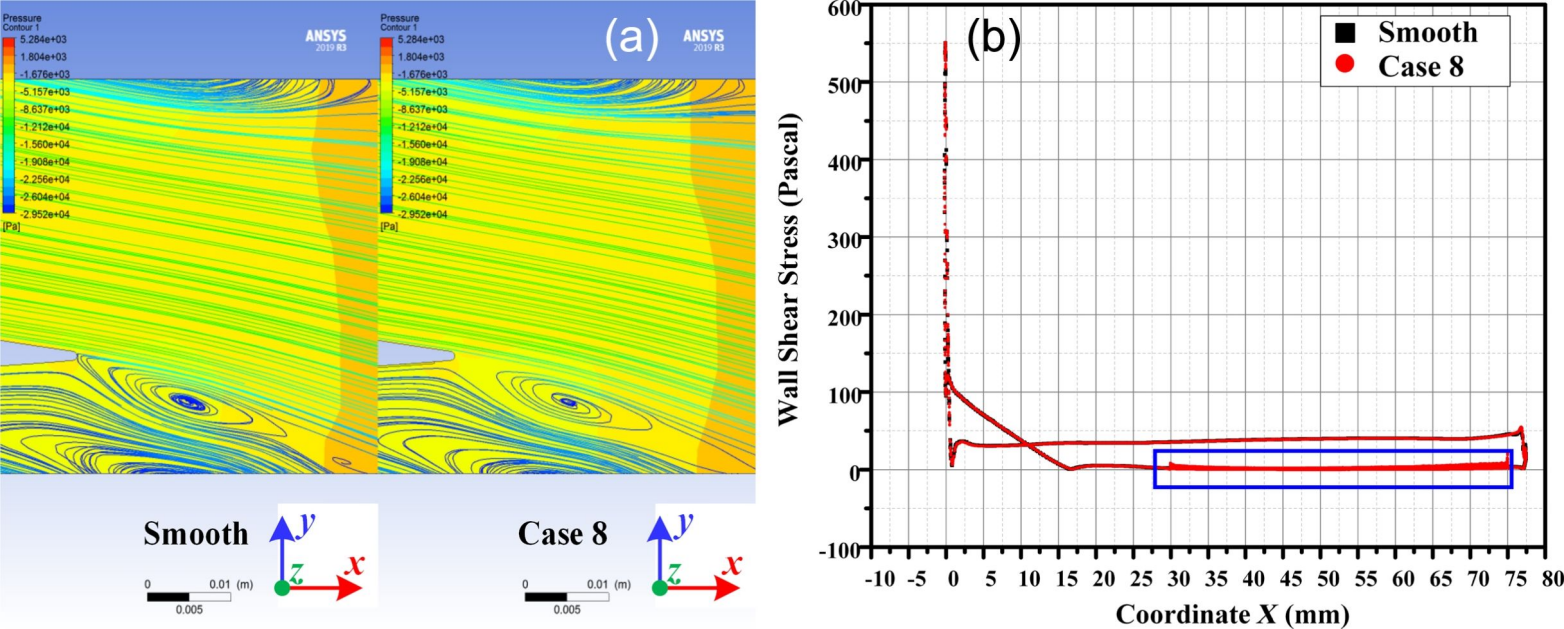
The effect of microtexture on (a) turbulent vortex and (b) overall shear stress distribution. Figure 17a used courtesy of ANSYS, Inc.

The influence of the microtexture on turbulent vortices is shown in Figure 17a; the contour shows the pressure distribution in the flow domain. The periodic boundary was used in the two pictures as the streamline release entrance, with identical streamline. It can be seen from the streamline that the microtexture effectively inhibits turbulence generation and reduces system energy consumption. Weakening of turbulences will cause a reduction of wall shear stress, which is reflected in the reduction of friction resistance.

The shear stress distribution on the smooth blade and the microtextured blade is shown in Figure 17b; the blue mark indicates that the placement of microtextures does not change the overall shear stress distribution of the blade. Instead, it generates shear stress fluctuations within the microtextured area.

### Wind tunnel experiment with the microtextured blade surface

#### Surface quality analysis of the microtextured blade

**Figure 18:**
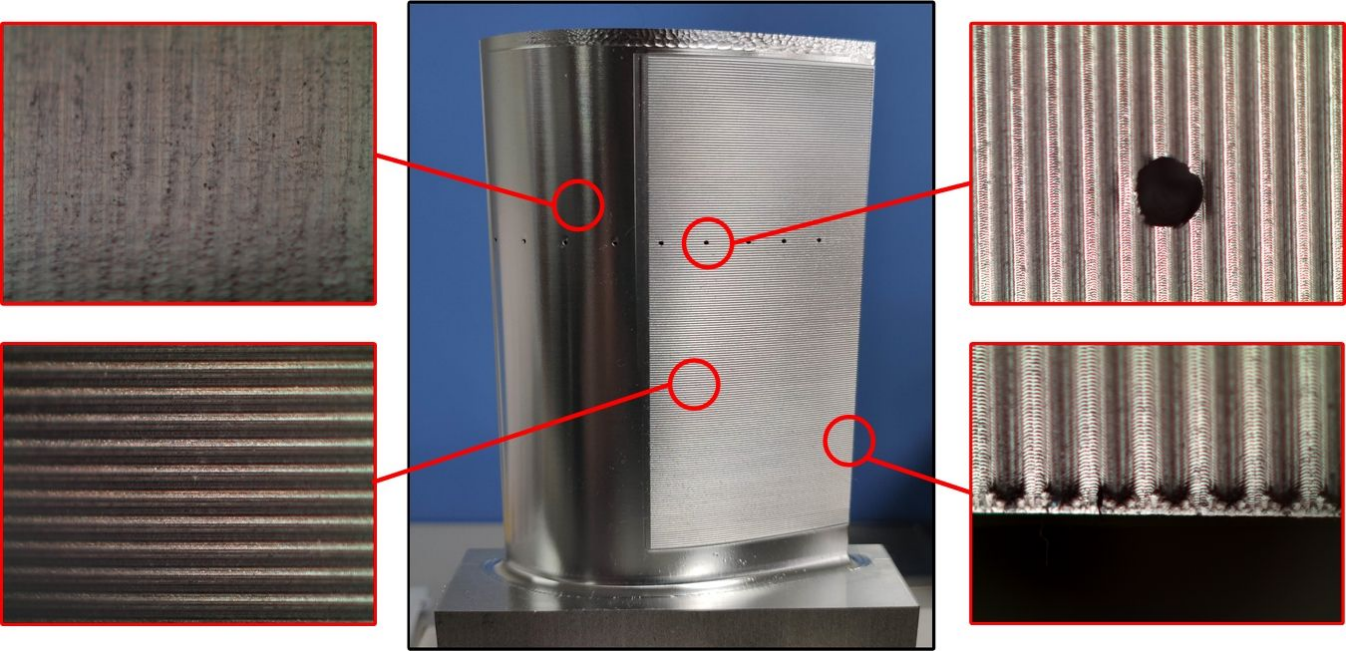
The processed blade with microtexture.

**Figure 19:**
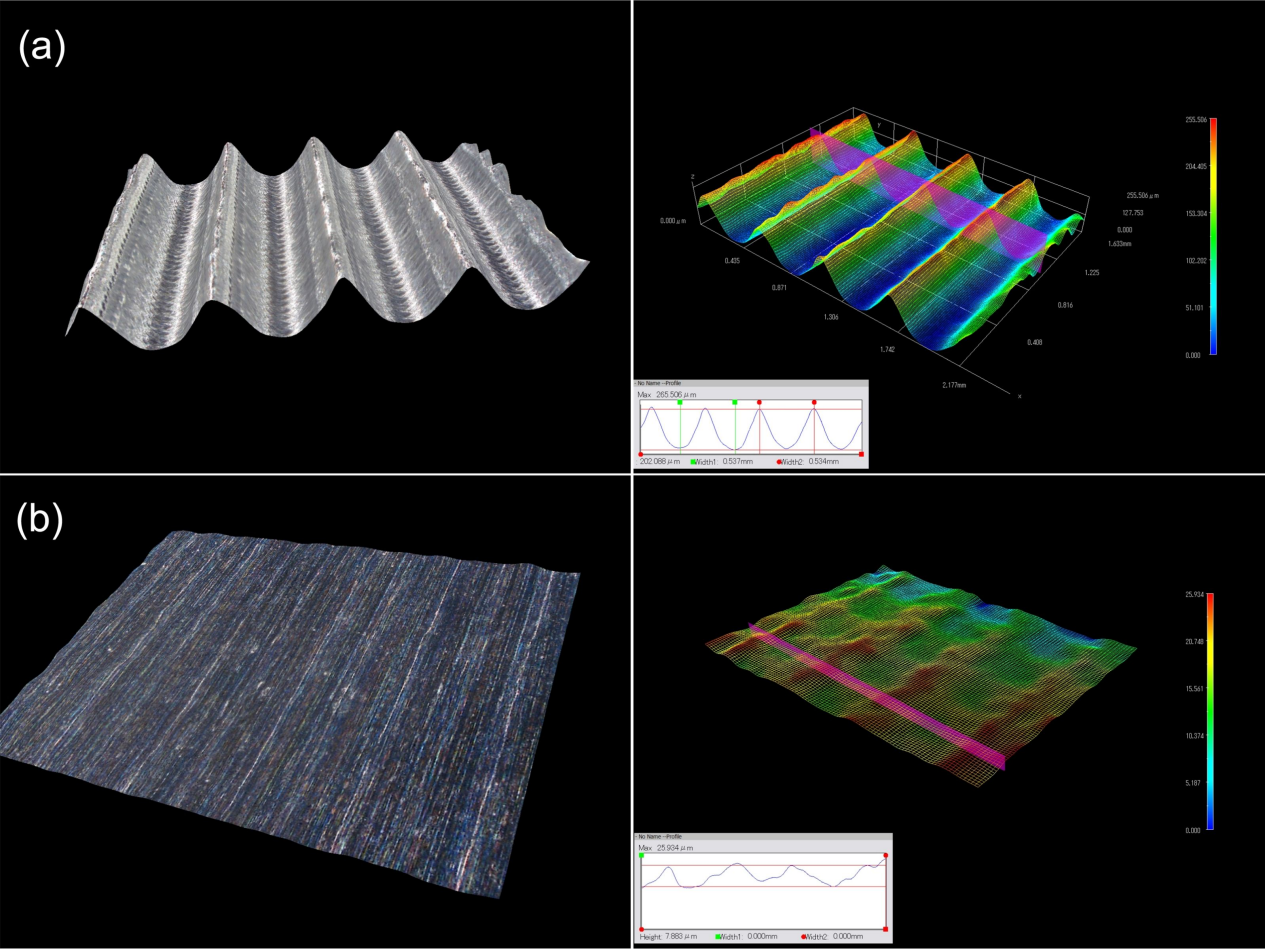
Microscopy observation of (a) microribs surface morphology and (b) blade surface morphology.

The processed blade, composed of 7075 series aluminum alloy, is displayed in Figure 18. The blade surface quality was assessed using a JSZ6S trinocular stereo microscope; the results showed that the processed blade has high quality and no obvious defects. In order to further analyze the processing quality, a HIROX KH-7700 three-dimensional video microscope was used to examine the microtexture and blade surface morphology, as shown in Figure 19.

**Figure 20:**
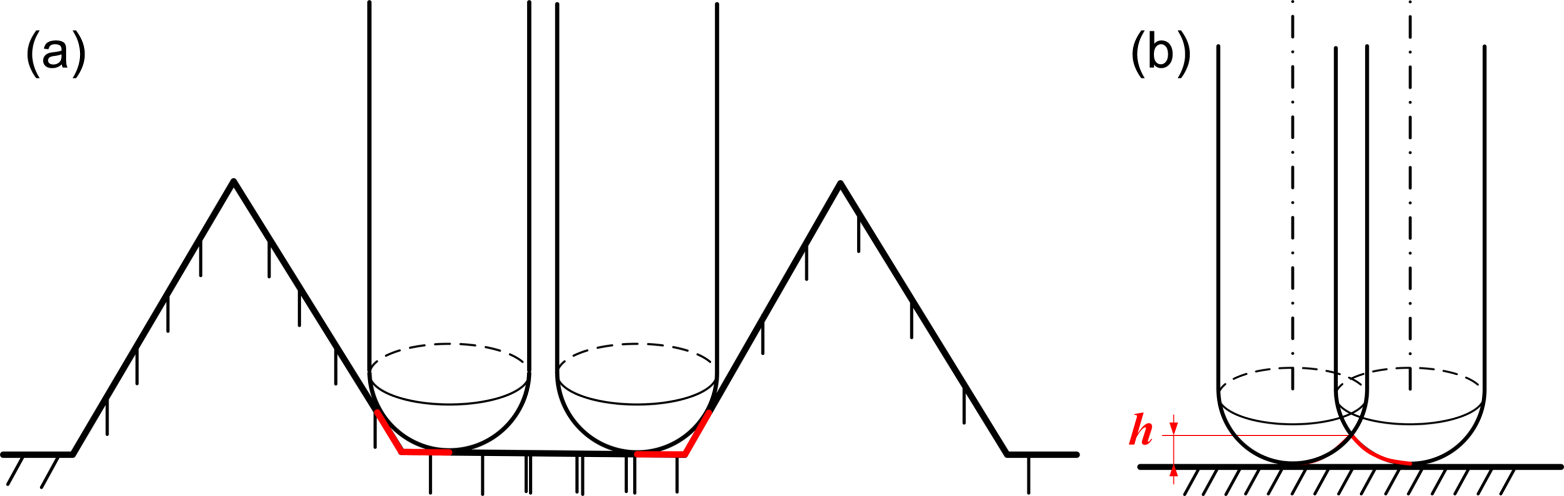
Machining error analysis diagram of (a) the microtexture and (b) plane processing.

The rib surface morphology and dimensional data are shown in Figure 19a. The theoretical height is 0.2 mm, the width is 0.3 mm, the spacing is 0.2 mm, and the rib–tip spacing is 0.5 mm. In contrast, the actual height measures are 0.202 mm, and the actual rib–tip spacing is 0.534 mm. The minimal machining error is due to the utilization of a ball end milling tool with a diameter of 0.2 mm, which has a processing residue at the corner of the bottom rib area (Figure 20a). Figure 19b indicates slight height fluctuations on the surface of the smooth blade, reaching a maximum deviation of 0.007 mm. This can be attributed to the point contact nature of the ball tip tool during the machining process and the spacing between tool paths. Thus, the machining coverage rate does not reach 100%, resulting in a residual height *h* as shown in Figure 20b. To sum up, the microtexture here meets the quality requirements.

#### Results of the experiments

The experimental results are given in Table 11 and Table 12.

**Table 11:** Experimental results at inlet and outlet of the smooth blade.

Angle of attack (°)	*TP*_1_ (Pa)	*P*_1_ (Pa)	*TP*_2_ (Pa)	*P*_2_ (Pa)	*V*_1_ (m/s)^a^	*V*_2_ (m/s)^b^	ξ_0_ (%)

52.8	108056	98763	105010	100920	123.66	91.56	41.61
54.8	109091	98833	104266	101071	129.50	69.00	58.98
57.0	111402	99905	104647	101292	135.91	70.21	65.45

^a^inlet velocity; ^b^outlet velocity; ^c^energy loss coefficient of the smooth blade.

**Table 12:** Experimental results at inlet and outlet of the microtextured blade.

Angle of attack (°)	*TP*_1_ (Pa)	*P*_1_ (Pa)	*TP*_2_ (Pa)	*P*_2_ (Pa)	*V*_1_ (m/s)^a^	*V*_2_ (m/s)^b^	ξ_1_ (%)^c^

52.8	108840	99248	105190	101131	125.23	79.23	46.17
54.8	109810	99393	104571	101269	130.10	69.38	60.09
57.0	110853	99359	104828	101488	136.24	69.87	63.02

^a^inlet velocity; ^b^outlet velocity; ^c^energy loss coefficient of the microtextured blade.

The results in Table 11 indicate that ξ_0_ rises with increasing angle of attack. The simulation reveals distinct phenomena occurring at three angles of attack; the separation phenomenon and vortex at the back section of the blade become more apparent and intense with the increase of the angle. Table 12 shows that the textured blade has a more significant effect on reducing ξ_1_ as the flow angle increases. At the flow angle of 57°, η_ξ_ = −3.7% based on Equation S5 of Supporting Information File 1, which indicates that the microtexture reduces energy consumption and improves the overall aerodynamic performance of the blades.

**Figure 21:**
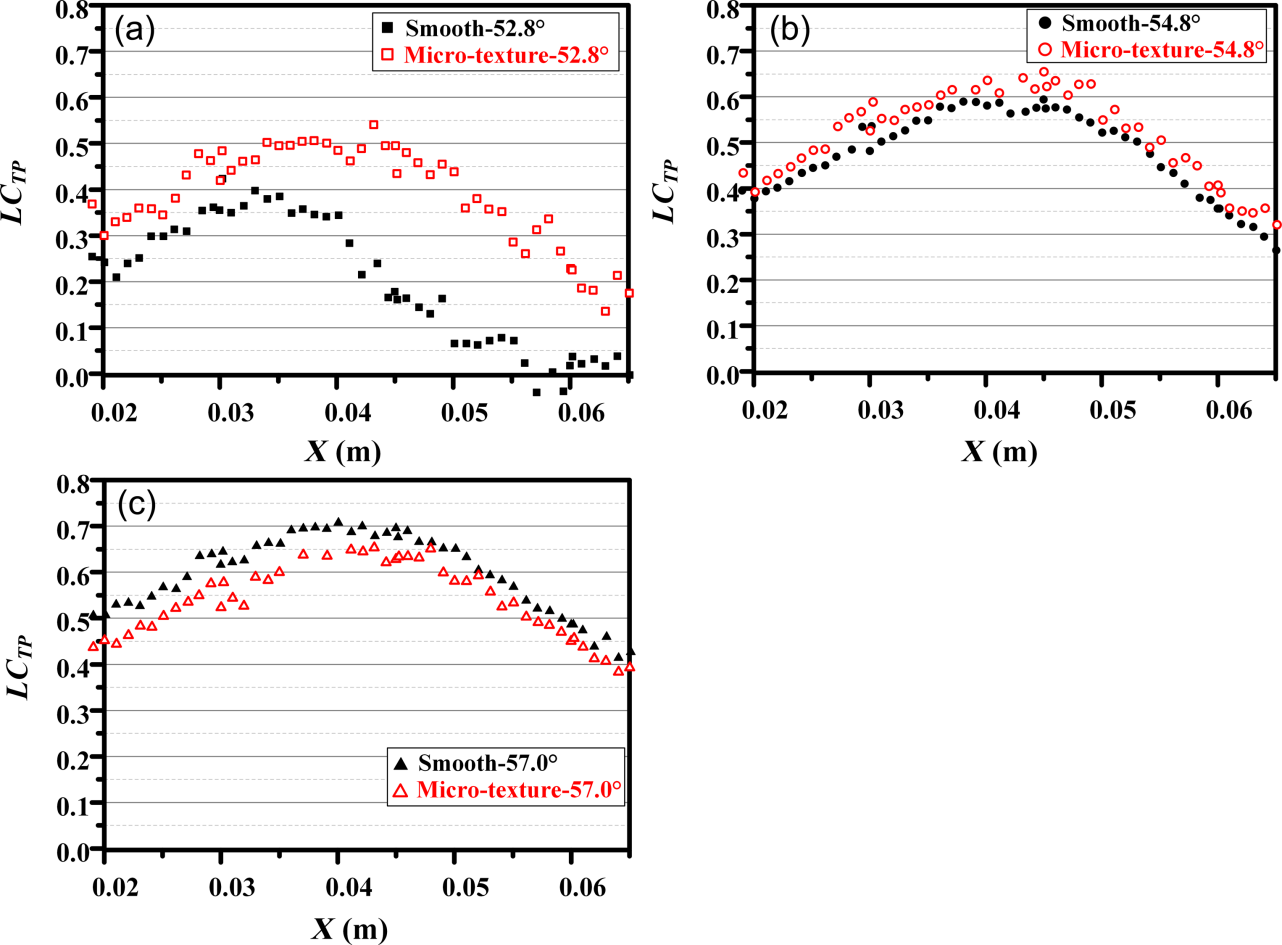
Distribution of *LC_TP_* of the single flow channel at angles of attack of (a) 52.8°, (b) 54.8°, and (c) 57.0°.

**Figure 22:**
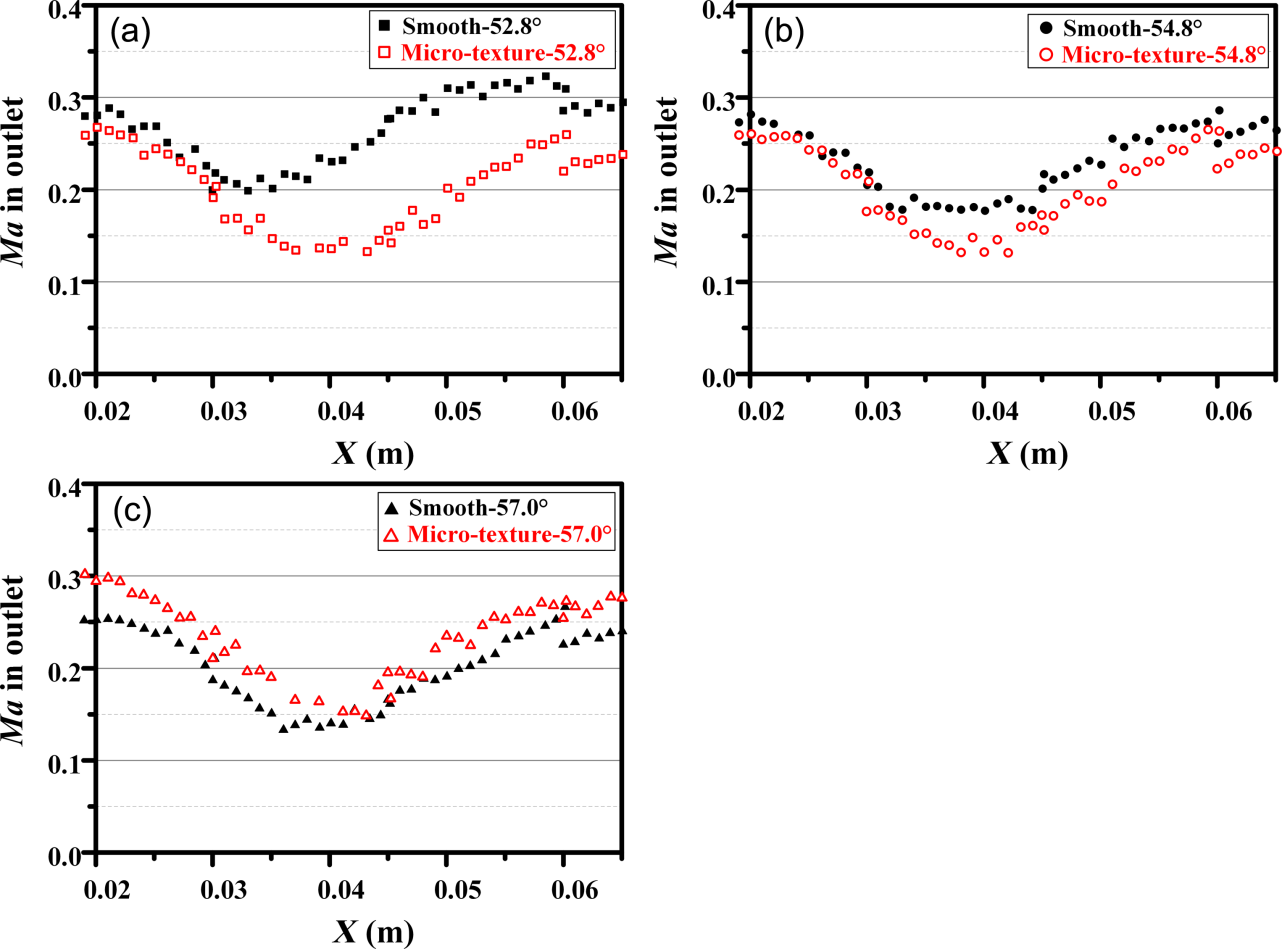
Distribution of Ma in the outlet of the single flow channel at angles of attack of (a) 52.8°, (b) 54.8°, and (c) 57.0°.

Figure 21 and Figure 22 illustrate the distribution of *LC_TP_* and outlet Ma at three different angles of attacks. A higher *LC_TP_* and a lower Ma indicate a poorer aerodynamic performance of the blade. According to Figure 21a and Figure 22a, the blade with microtexture exhibits an increase in *LC_TP_* from 0.4 to 0.5, while the Ma in the flow channel center decreases from 0.2 to 0.13. These results indicate that, at the flow angle of 52.8°, the microtexture has an adverse effect on the aerodynamic performance of the blade, resulting in increased drag. Figure 21c and Figure 22c show that the blade with microtexture yields a smaller *LC_TP_* compared to the smooth blade, and the Ma is slightly higher. These results indicate a reduction in system energy loss. Overall, the microtexture arranged in the back section of the blade positively impacts aerodynamic performance and reduces system energy loss, particularly at the angle of attack of 57°.

**Figure 23:**
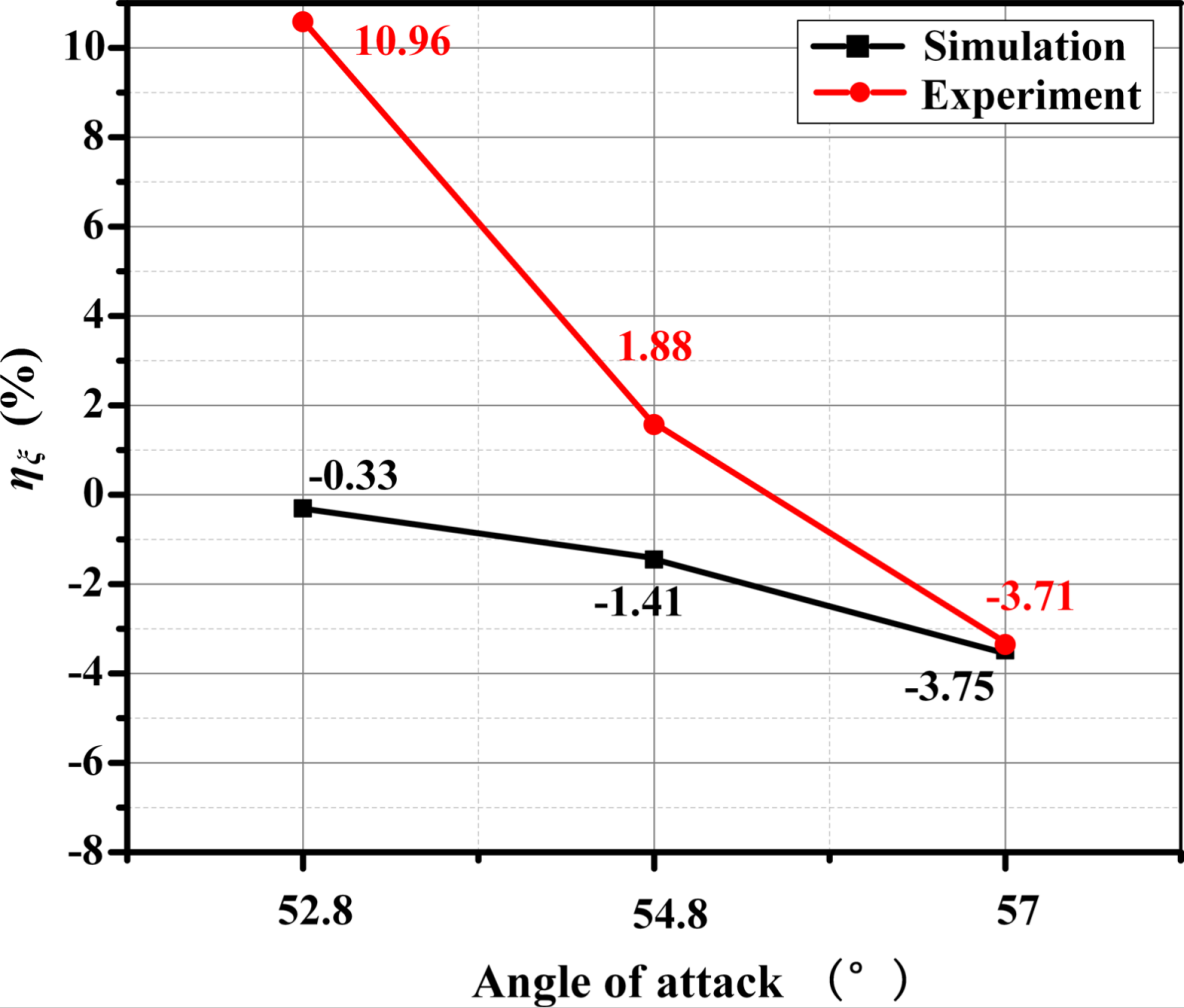
Comparison between the simulation value and the experimental value of η_ξ_.

The microtexture was arranged at the back end of the blade suction surface based on the analysis of the simulation results, and the drag reduction effect of the microtexture was verified in the wind tunnel experiment. As shown in Figure 23, the drag reduction performance of the microtexture blade is the best when the angle of attack is 57°; η_ξ_ in the experiment can reach −3.7%. Although the difference between the simulation results and the experimental results is large under the other two attack angles, the trend of η_ξ_ of the two results is the same. The larger the angle of attack, the smaller η_ξ_.

## Conclusion

This paper studies an axial flow compressor and presents a simplified numerical simulation method for the rotating blade surface. Furthermore, microtexture design and simulation analysis are carried out on the blade surface to explore the drag reduction performance and mechanism of microtexture. The conclusions are as follows: (1) A simplified simulation method is proposed from the whole impeller to a single impeller blade, establishing the relationship between plane and surface. Theoretical calculations and numerical simulations are employed to design and verify the optimal microtexture for drag reduction performance. The determined microtexture dimensions are a height of 0.2 mm, a width of 0.3 mm, and a spacing of 0.2 mm. (2) The drag reduction mechanism is analyzed and compared for microtextures with different geometric size factors. The presence of microtextures on the blade surface effectively impedes turbulence generation, thus, reducing the turbulent kinetic energy and wall shear stress to reduce drag. (3) The simulation results reveal that positioning the optimally sized microstructure at the back end of the blade yields significant benefits. The DRR for a single blade reaches 1.31%, accompanied by a reduction of 1.45% in η_ξ_. (4) A blade cascade experiment is conducted in the high-speed wind tunnel to analyze the energy loss coefficient and wake loss distribution. The results demonstrate a reduction in energy consumption of 3.7% at a flow velocity of 136.24 m/s and an attack angle of 57°.

Bionic microstructures have little influence on the overall strength of the objects they are attached to because of their small size. Their particular functions are of high research value in the application of object surfaces, but there are also some challenges in practical applications. The cost of microstructures in large-area manufacturing and application is large. However, the size effect is the key of microstructures exhibiting good performance. Hence, the large-area manufacturing of high-precision microstructures is worth studying. Chemicals (e.g., polydimethylsiloxane) can quickly replicate biomimetic microstructures, but the operation process is complex, and the soft surfaces are not suitable for surfaces in high-speed flows.

## Supporting Information

Supporting information text contains the hydrodynamic theory covered in this paper, including the boundary layer theory, the formulas for calculating the drag reduction performance of the blade and a description of flow separation on the blade surface.

File 1 Boundary layer theory, drag reduction formulas, and blade surface flow.

## Data Availability

The data that supports the findings of this study is available from the corresponding author upon reasonable request.
