# Supplementary material for: Investigation on drag reduction on rotating blade surfaces with microtextures
Source: Beilstein J Nanotechnol. 2024 Jul 10;15:833–53. doi: 10.3762/bjnano.15.70 (PMC11252563; doi:10.3762/bjnano.15.70)
Supplement: File 1 — Boundary layer theory, drag reduction formulas, and blade surface flow. [file Beilstein_J_Nanotechnol-15-833-s001.pdf]

BEILSTEIN JOURNAL OF NANOTECHNOLOGY

Supporting Information

for

Investigation on drag reduction on rotating blade surfaces with microtextures

Qinsong Zhu, Chen Zhang, Fuhang Yu and Yan Xu

Beilstein J. Nanotechnol. **2024,** *15,* 833–853. doi:10.3762/bjnano.15.70

Boundary layer theory, drag reduction formulas, and blade surface flow

License and Terms: This is a supporting information file under the terms of the Creative Commons Attribution License (https://creativecommons.org/licenses/by/4.0). Please note that the reuse, redistribution and reproduction in particular requires that the author(s) and source are credited and that individual graphics may be subject to special legal provisions.
The license is subject to the *Beilstein Journal of Nanotechnology* terms and conditions: (https://www.beilstein-journals.org/bjnano/terms)

Boundary layer theory

As shown in Figure 1, the fluid near the wall at the front end of the flat plate is laminar. However, the flow in the boundary layer will gradually transform to turbulent as the fluid flows along the flat plate. This transformation occurs gradually, and there is no distinct boundary line between the laminar and turbulent flow regions.

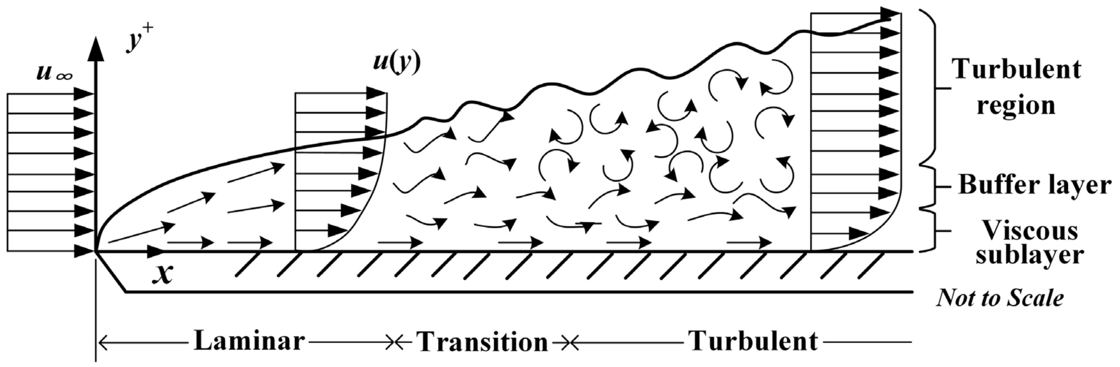

Figure S1: Development of the boundary layer on near wall surface.

The near-wall region within the boundary layer is the primary source of turbulent kinetic energy. This region can be divided into three distinct regions, namely, the viscous sublayer, the buffer layer, and the turbulent region (log-law region) [1]. The position of each region within the boundary layer is determined based on the dimensionless normal distance from the wall [2-4]:

$$y^+ = \frac{yu_\tau}{v}, \tag{S1}$$

where y is the distance from the wall, u_τ is the wall stress shear velocity, and v is the kinematic viscosity of the fluid [5]. According to Figure 2, the near-wall

area is usually in the range of $y^+ \leq 100$. The range of the viscous sublayer region is $0 \leq y^+ \leq 5$; here, the viscous shear stress is dominant, and the turbulent shear stress is zero. The buffer layer is $5 \leq y^+ \leq 30$, characterized by the simultaneous presence of both viscous and turbulent shear stresses. The overlap layer ranges from $30 \leq y^+ \leq 100$, where the turbulent shear stress becomes dominant [6].

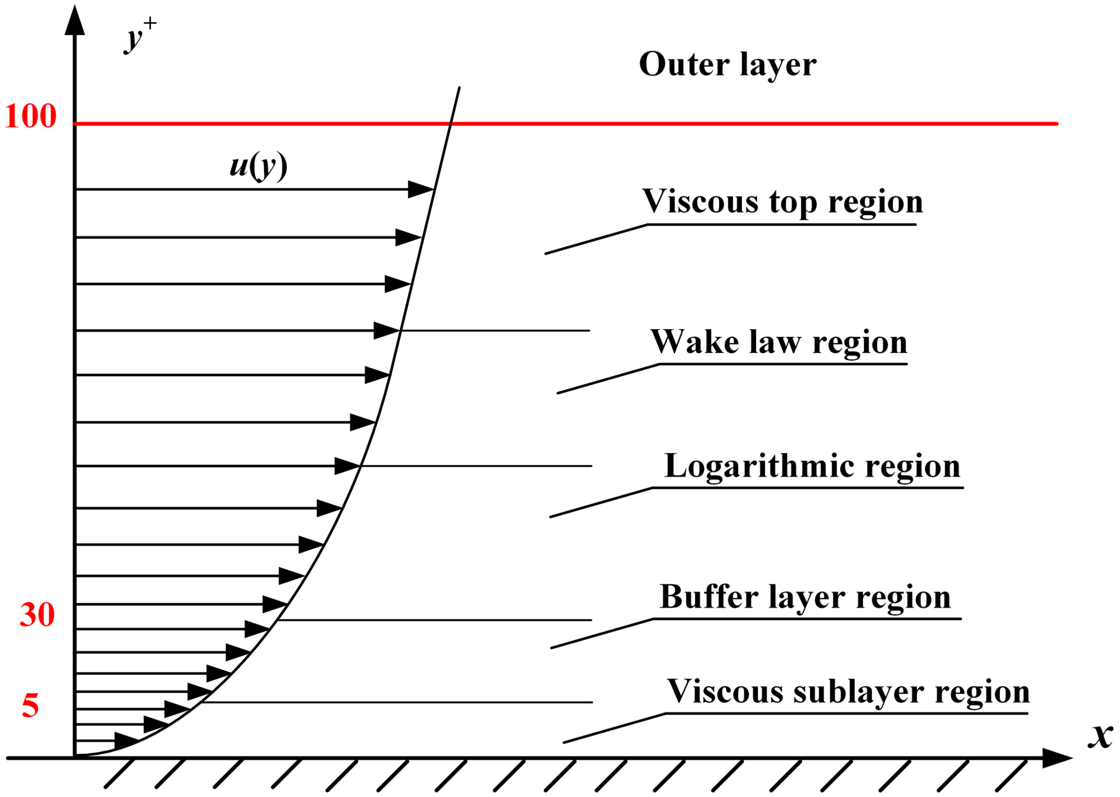

Figure S2: The range of y^+ corresponding to each region in the boundary layer.

The turbulence within the boundary layer forms a fluid structure called "coherent structure", which can be divided into different types, including vortex, blast, and sweep [7]. Robinson [8] proposed an idealized schematic diagram of vortex structures in different regions of the turbulent boundary layer. The flow vortex

is prominent in the buffer sublayer, while the hairpin vortex prevails in the outer layer. These two types of vortices overlap within the logarithmic region.

The velocity gradient in the vicinity of the wall intensifies because of the vortices, thereby, generating a transverse shear force that contributes to heightened resistance. Thus, the control of vortices becomes advantageous in achieving a surface with reduced resistance. By analyzing the behavior of vortices, the design of microtextures aims to facilitate vortex uplift, reducing shear stress and attaining a surface exhibiting lower resistance.

Drag reduction formulas

The total resistance (F) of the blade is mainly composed of two parts, that is, pressure drag (F_p), caused by the pressure gradient, and frictional drag (F_f), caused by the viscous of the wall. Their relationship is as follows:

$$F = F_p + F_f, \tag{S2}$$

$$F_f = C_f \frac{\rho u_0^2}{2} A_f, \tag{S3}$$

where C_f is the friction coefficient of the microstructure, A_f is the surface area of the object, ρ is the density of the fluid, and u_0 is the velocity of the fluid. Thus, the drag reduction rate (DRR) of the blade surface can be expressed as:

$$\mathrm{DRR} = \frac{F_1 - F_0}{F_0} \times 100\%, \tag{S4}$$

where F_0 and F_1 are the total resistance on the smooth blade and microtextured blade, respectively. Then the change rate of energy loss coefficient (η_ξ) and

total pressure loss coefficient (LC_{TP}) are used to represent the change of resistance of the blade and flow path to improve the reliability of the results [9]:

$$\eta_{\xi} = \frac{\xi_1 - \xi_0}{\xi_0} \times 100\%, \tag{S5}$$

where ξ_0 and ξ_1 are the energy loss coefficient of the smooth blade and microtextured blade, respectively. The energy loss coefficient (ξ) is defined as:

$$\zeta = \frac{S_I - S_A}{S_I} = \frac{\left(P_2/TP_2\right)^{\frac{k-1}{k}} - \left(P_2/TP_1\right)^{\frac{k-1}{k}}}{1 - \left(P_2/TP_1\right)^{\frac{k-1}{k}}}, \tag{S6}$$

where S_I and S_A are the static enthalpy of the isentropic process and actual process, respectively; TP_1 and TP_2 are the total pressure on the inlet and outlet of the impeller flow path, respectively; P_1 and P_2 are the static pressure on the inlet and outlet of the impeller flow path, respectively; k is the specific heat ratio and, for air, it is 1.4. LC_{TP} can be expressed as:

$$LC_{TP} = \frac{TP_2 - TP_1}{TP_1}. \tag{S7}$$

TP is equal to the sum of dynamic pressure (DP) and P, which can be calculated by the following equation:

$$TP = P\left[1 + \mathrm{Ma}^2\left(\frac{k-1}{2}\right)\right]^{\frac{k}{k-1}}, \tag{S8}$$

where Ma is the Mach number; Ma is defined as:

$$\mathrm{Ma} = V/C, \tag{S9}$$

where V is the airflow velocity, and the speed of sound, C, is defined as:

$$C = \sqrt{kTR}, \tag{S10}$$

where T is absolute temperature, and R is the specific gas constant. In this paper, the value of T and R are 300 K and 287 J·kg^{-1}·K^{-1}, respectively.

η_ξ represents the change of energy consumption of the whole system. A higher η_ξ indicates an increase of energy consumption of the system, indicating that microtexture has an adverse effect on the aerodynamic performance of the blade.

Flow separation on the blade surface

The blade is a curved surface with a complicated flow situation, and flow separation occurs at high-speed air flow over the blade. Figure 3 shows the schematic of flow around the airfoil surface, where point A is the stagnation point, point B is the highest point, and point C is the flow separation point. The region between A and B is the pressure surface, characterized by smooth airflow along the wall without boundary layer separation. Therefore, this region is discretized and treated as a collection of small local planes. The region between B and C is the suction surface where the flow surface increases, causing the outflow to decelerate, the pressure to rise, and the kinetic energy to be converted into pressure energy. Conversely, the pressure difference behind point C triggers flow reversal and separation of the boundary layer from the wall, resulting in a vortex zone known as the separation region. The generation of the separation region significantly impacts the outflow boundary, thereby relieving the influence of viscosity on the thin fluid layer near the wall.

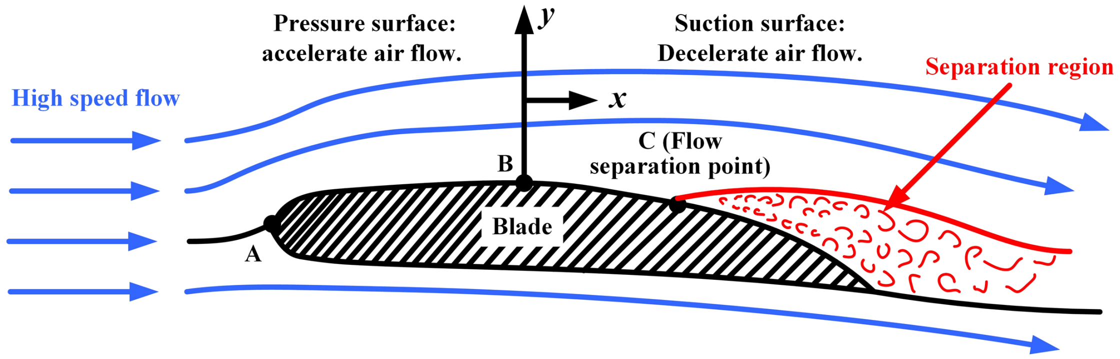

Figure S3: The schematic diagram of boundary layer separation on airfoil surface**.**

References

1. Bradshaw, P.; Huang, G. P. *Proc. R. Soc. London, Ser. A* **1995,** *451,* 165–188. doi:10.1098/rspa.1995.0122
2. Spalding, D. B. *J. Appl. Mech.* **1961,** *28,* 455–458. doi:10.1115/1.3641728
3. Kline, S. J.; Reynolds, W. C.; Schraub, F. A.; Runstadler, P. W. *J. Fluid Mech.* **1967,** *30,* 741–773. doi:10.1017/S0022112067001740
4. Robinson, S. K. *Annu. Rev. Fluid Mech.* **1991,** *23,* 601–639. doi:10.1146/annurev.fl.23.010191.003125
5. Zhu, W.; Bons, J.; Gregory, J. *Exp. Fluids* **2023,** 64. doi:10.1007/s00348-022-03550-2
6. White, F. *Eur. J. Mech., B: Fluids* **2005,** *20,* 157–158. doi:10.1177/0115426505020001157

7. Hon, T.-L.; Walker, J. D. *An Analysis of the Motion and Effects of Hairpin Vortices;* Lehigh University, Bethlehem PA, USA,1987.
8. Robinson, S. K. A Review of Vortex Structures and Associated Coherent Motions in Turbulent Boundary Layers. In *Structure of Turbulence and Drag Reduction;* Zurich, Switzerland, July 25–28, 1989; Gyr, A., Ed.; Springer, 2012. doi:10.1007/978-3-642-50971-1_2
9. Denton, J. D. Loss mechanisms in turbomachines. In *ASME 1993 International Gas Turbine and Aeroengine Congress and Exposition;* Cincinnati, OH, USA, May 24–27, 1993; American Society of Mechanical Engineers; 1993. doi:10.1115/93-GT-435
